# Fluorescent base analogues in gapmers enable stealth labeling of antisense oligonucleotide therapeutics

**DOI:** 10.1038/s41598-021-90629-1

**Published:** 2021-05-31

**Authors:** Jesper R. Nilsson, Tom Baladi, Audrey Gallud, Dženita Baždarević, Malin Lemurell, Elin K. Esbjörner, L. Marcus Wilhelmsson, Anders Dahlén

**Affiliations:** 1 Department of Chemistry and Chemical Engineering, Chalmers University of Technology, 412 96 Gothenburg, Sweden; 2 Medicinal Chemistry, Research and Early Development, Cardiovascular, Renal and Metabolism (CVRM), BioPharmaceuticals R&D, AstraZeneca, Gothenburg, Sweden; 3 Department of Biology and Biological Engineering, Chalmers University of Technology, 41296 Gothenburg, Sweden; 4 Bioscience, Research and Early Development, Cardiovascular, Renal and Metabolism (CVRM), BioPharmaceuticals R&D, AstraZeneca, Gothenburg, Sweden; 5 Oligonucleotide Discovery, Discovery Sciences, BioPharmaceuticals R&D, AstraZeneca, Gothenburg, Sweden; 6 Present Address: Oligonucleotide Discovery, Discovery Sciences, BioPharmaceuticals R&D, AstraZeneca, Gothenburg, Sweden; 7 Present Address: Advanced Drug Delivery, Pharmaceutical Sciences, R&D, AstraZeneca, Gothenburg, Sweden

**Keywords:** Imaging, Microscopy, Sensors and probes, Drug discovery, Chemistry

## Abstract

To expand the antisense oligonucleotide (ASO) fluorescence labeling toolbox beyond covalent conjugation of external dyes (*e.g.* ATTO-, Alexa Fluor-, or cyanine dyes), we herein explore fluorescent base analogues (FBAs) as a novel approach to endow fluorescent properties to ASOs. Both cytosine and adenine analogues (tC, tC^O^, 2CNqA, and pA) were incorporated into a 16mer ASO sequence with a 3-10-3 cEt-DNA-cEt (cEt = constrained ethyl) gapmer design. In addition to a comprehensive photophysical characterization, we assess the label-induced effects on the gapmers’ RNA affinities, RNA-hybridized secondary structures, and knockdown efficiencies. Importantly, we find practically no perturbing effects for gapmers with single FBA incorporations in the biologically critical gap region and, except for pA, the FBAs do not affect the knockdown efficiencies. Incorporating two cytosine FBAs in the gap is equally well tolerated, while two adenine analogues give rise to slightly reduced knockdown efficiencies and what could be perturbed secondary structures. We furthermore show that the FBAs can be used to visualize gapmers inside live cells using fluorescence microscopy and flow cytometry, enabling comparative assessment of their uptake. This altogether shows that FBAs are functional ASO probes that provide a minimally perturbing in-sequence labeling option for this highly relevant drug modality.

## Introduction

The seminal work on antisense technology emerging in the late 1970s^1,2^, along with the advancement in fundamental understanding of the regulatory processes of ribonucleic acids (RNAs) occurring over the past two decades, have set the stage for therapeutic intervention at the translational level^3–5^. This has enabled the development of several novel oligonucleotide-based (ON) therapeutics, such as small interfering RNAs (siRNAs) and antisense oligonucleotides (ASOs), capable of modulating the expression of disease-associated genes^6,7^. A particularly attractive ASO design, owing to its catalytic mode of action, reasonably straightforward design, and potential for gymnotic delivery, is the gapmer^4,8^. Gapmers are typically single-stranded, short (16–20 nucleotides (nt)), synthetic ASOs with an 8–10 nt DNA stretch in the middle of the sequence, termed “the gap”, which is flanked on both sides by “wings” consisting of 1–5 modified nucleotides, which provide improved nuclease stability and additional RNA affinity. Gapmers induce their pharmacological effect by associating to the complementary RNA target through Watson–Crick base-pairing. This initiates recruitment of endonuclease RNase H1, which selectively cleaves the RNA strand of the hybrid duplex, allowing the intact gapmer to continue its catalytic cycle. In addition to the recently developed RNA vaccines for combating the SARS CoV-2 pandemic, a total of ten ON drugs have been approved by the U.S. Food and Drug Administration and/or the European Medicines Agency^9^. Gapmers currently constitute a majority of the approved therapeutics on this list, which underlines their importance in this field.

Fluorescence-based techniques are invaluable tools for direct and detailed visualization of dynamic biomolecular interactions in live cells and tissue. This has enabled studies of several aspects that are central to ASO development, including cellular uptake, trafficking, and protein interactions^10–12^. The currently favored approach for introducing fluorescent labels to ASOs is by covalent conjugation of fluorophores, such as ATTO-, Alexa Fluor-, or cyanine (Cy) dyes, to the end of the oligonucleotide. Although this external end-labeling scheme typically renders the gapmer highly emissive, the significant size, amphiphilicity, and/or additional charges associated with the fluorophores can be problematic as they may affect properties that are critical to the gapmer’s therapeutic performance, including the affinity to the target RNA and/or interactions with RNase H1, and may also affect the affinity to lipid structures. Label-induced perturbations of the properties of therapeutic oligomers have indeed been reported^13–15^. Edelson et al*.* for instance, showed that the propensity of DNA-targeting oligomer-fluorophore conjugates to enter cell nuclei varied substantially depending on the identity of the fluorophore and characteristics of the linker chemistry^15^. Furthermore, affinity studies of short Cy5-labeled DNAs have shown that the presence of the fluorophore, which in the case of Cy5 is one of the most widely used for ASO labeling, affects the thermodynamic stability of the resulting DNA duplex^16,17^. There have also been accounts that highlight the need to consider lipophilicity when selecting a fluorescent label, by showing that the lipid bilayer affinity of common water-soluble dyes can differ dramatically^18^.

A conceptually different approach to achieve fluorescent ASOs would be to incorporate fluorescent base analogues (FBAs) as an integral part of the oligonucleotide structure. FBAs are synthetic nucleobases typically designed to be both structural and functional analogues of their canonical counterpart, with the added value of being significantly fluorescent^19–21^. Therefore, FBA-containing oligonucleotides largely retain their native base-pairing, duplex stability, and secondary structure without introducing significant steric bulk to the oligomer. A unique and attractive feature inherent to FBA labeling is that the fluorophore is firmly located in the oligomer stack, which allows for positioning of the label at a specific site of interest with a level of spatial control that is not attainable with external fluorophores. FBAs have previously been employed as oligomer-incorporated reporters to study DNA duplex-^22^, triplex-^23^, and G-quadruplex^24^ formation, as well as in Förster resonance energy transfer (FRET) applications to monitor ligand binding^25,26^, duplex form transitions^27,28^, and protein-nucleic acid interactions^29,30^. These studies and others have provided important novel insights on isolated biological systems, while there is a notable lack of FBA applications in more complex biological matrixes. In fact, the first report where an FBA was used to achieve fluorescent properties in live cells was recently published by us, where we demonstrated the applicability of the cytosine analogue tC^O^ for studying delivery of functional mRNA to human cells^31^.

**Figure 1:**
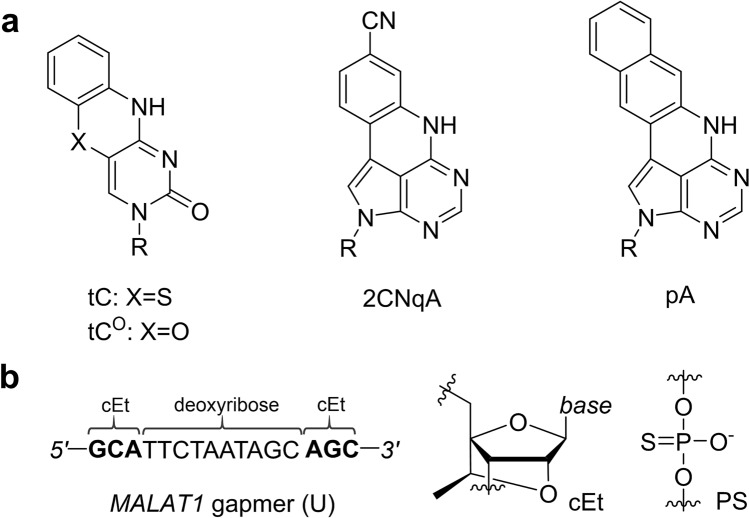
(**a**) Two fluorescent base analogues from the tricyclic cytosine family (tC and tC^O^) and two adenine analogues (2CNqA and pA) were used in this study; R = deoxyribose. (**b**) Sequence and chemistry of the *MALAT1*-targeting gapmer (U) explored in this study. The three flanking bases on each side (wings, bold font) have constrained ethyl (cEt) sugars, all other sugars are deoxyribose. The nucleotides in the gapmer are linked by phosphorothioate (PS) groups.

In this work, we continue our development of FBAs as internal fluorescent labels in ON therapeutics by exploring them as an alternative way of achieving fluorescent ASOs, with the added value of in-sequence positioning with maintained canonical base-pairing. We perform a comprehensive photophysical characterization, which is essential for accurate interpretation of fluorescence-based data (*e.g.* from live cell microscopy studies), and study how different FBAs, and positions in the gapmer sequence, affect their RNA target affinities, secondary structures, knockdown activities, and uptake characteristics. The study includes the tricyclic cytosine analogues tC^32^ and tC^O^^33^, as well as the more recently developed quadracyclic adenine analogue 2CNqA^34^, and pentacyclic adenine analogue pA^35^ (Fig. 1a), all of which were incorporated into a 16 nt gapmer sequence (Fig. 1b) targeting the long non-coding RNA *Metastasis Associated Lung Adenocarcinoma Transcript 1* (*MALAT1*)^36^.

The FBAs included in this work have previously been characterized as monomers^33,35,37–39^, as well as inside RNA^34,40^ and/or DNA^32,33,41,42^, where they exhibit excellent base-mimicking and emissive properties. *MALAT1* is ubiquitously expressed and highly abundant in the nucleus; it is involved in multiple pathological processes, including tumor progression and metastasis, and has consequently been targeted for silencing by several ASO therapies, including gapmers^43^ and gapmer conjugates^44–46^. The gapmer sequence and design used here (Fig. 1b) were developed in a previous study^46^ and the modifications, *i.e.* the 2’-4’-ethylenoxy-bridged ribose (or *constrained ethyl*, cEt) wings and phosphorothioate (PS) linkages, are widely used in the field to improve ASO stability, potency, and bioavailability^47,48^. In this work, we show that the combined base-mimicking and emissive properties of the investigated FBAs, as established in RNA and/or DNA, largely extends to the gapmer environment, and demonstrate their usefulness as fluorescent probes in live cells using fluorescence microscopy and flow cytometry. The FBA labeling approach thus opens new avenues for studying ASOs and provides a powerful alternative to external labels for fluorescence-based investigations where a native behavior is key.

## Material and methods

### Oligonucleotide synthesis

Gapmers were synthesized on a 16 or 32 μmol scale on an ÄKTA Oligopilot 10 system using PS 5G UnyLinker support (GE Healthcare, 347 μmol/g). All DNA phosphoramidites were purchased from Sigma Aldrich. All cEt phosphoramidites were obtained from Pharmaron. The 5'-amino-modifier C6 was obtained from GlenResearch. FBA phosphoramidites were prepared as described in the literature (tC^49^, 2CNqA^34^, pA^35^) and/or acquired from GlenResearch (tC, tC^O^). Phosphoramidites were dissolved to a final concentration to 0.1 M (3 equivalents) in DNA-grade acetonitrile (ACN) prior to use. Detritylation was performed using 3 vol-% dichloroacetic acid in toluene. 5-(benzylthio)-1H-tetrazole (BTT) was used as activating agent (0.3 M in ACN) for the couplings. Recirculation times of phosphoramidites were 5 min for DNA building blocks, 10 min for 2’-cEt building blocks, and 40 min for the FBA and 5'-amino-modifier C6 building blocks. Phenylacetyl disulfide (PADS) was dissolved in a 50 vol-% solution of 3-picoline in ACN (0.2 M) and aged for 24 h before use. Equal volumes of Cap A (9.1 vol-% acetic anhydride in tetrahydrofuran (THF)) and Cap B (THF/*N*-methylimidazole/pyridine 80:10:10 vol-%) were mixed in situ for capping. Cyanoethyl backbone removal was performed with 20 vol-% diethylamine in ACN after a final 5’-detritylation. Gapmers were cleaved from the solid support and further deprotected by treatment with aqueous ammonia (26 mass-%) at 55 °C for 15–20 h and were subsequently purified using ion-pairing HPLC on reverse phase columns. The gapmers labeled with Cy3 were synthesized as above, with the exception that they were purified with the 5’-monomethoxytrityl (MMT) protecting group on. After removal of the MMT group, the free 5’-NH_2_ was reacted with NHS-activated 4-azidobutanoic acid and further conjugated to Cy3-dibenzocyclooctyne (Cy3-DBCO, Sigma) via strain-promoted azide-alkyne cycloaddition (SPAAC, Supplementary Fig. S14). HPLC and HRMS data for all synthesized gapmers are provided in Supplementary Figs. S1–S13 and S15–S16). UV purities were determined using ion-pairing LCMS and are stated at 260 nm. Yields are given based on the initial resin loading and gapmer content of the final product, as calculated from UV absorption.

### Spectroscopic characterization

#### Sample preparation and general information

The spectroscopic characterization was carried out at room temperature (RT, *ca.* 22 °C) in 10 mM RNase-free phosphate buffer (pH 7.4) containing 1.0 mM EDTA and 100 mM added NaCl. Concentrations of single-stranded gapmers and/or RNA were determined spectroscopically using the Lambert–Beer equation. The molar absorptivities at 260 nm, of single-stranded oligomers (ε_*O*_, unit: L[mol oligomer]^-1^ cm^-1^), were calculated based on nucleobase composition according to:

$${\varepsilon }_{O}(\lambda )= 0.9\times \sum {\varepsilon }_{N}(\lambda )$$

with each nucleotide contributing with the following molar absorption coefficients (ε_N_): adenine: 15,300 M^−1^ cm^−1^, thymine or uracil: 9300 M^−1^ cm–^−1^, guanine: 11,800 M^−1^ cm^−1^, cytosine 7,400 M^−1^ cm^−1^, tC: 13,500 M^−1^ cm^−1^, tC^O^: 11,000 M^−1^ cm^−1^, 2CNqA: 14,600 M^−1^ cm^−1^, pA: 22,300 M^−1^ cm^−1^. A factor of 0.9 was applied to account for the hypochromic base stacking effect. The concentration of the Cy3-labeled oligomer was determined using the molar absorptivity for the Cy3 band (ε_Cy3_ = 151,000 M^-1^ cm^-1^ at 553 nm, as provided by Sigma). Gapmer:RNA duplexes were afforded by first mixing the single-strands at RT, then annealing them in solution using the following temperature program: RT to 85 °C at 4 °C/min, remain at 85 °C for 15 min, 85 °C to RT at 1.5 °C/min.

#### UV–vis absorption spectra

Absorption spectra were recorded on a Cary 4000 or Cary 5000 (Varian Technologies) spectrophotometer using a 1.0 nm wavelength interval (spectral band width (SBW): 1–2 nm, integration time: 0.05–0.1 s, optical path length: 3.0 mm). All spectra were baseline corrected by subtracting the corresponding absorption from the solvent only.

#### Steady-state emission spectra

Emission spectra were recorded on a SPEX Fluorolog (Jobin Yvon Horiba) fluorimeter. Samples were excited at 345 nm (tC-2, tC-2^w^, tC-3), 350 nm (tC-1, tC^O^-1, tC^O^-2, 2CNqA-1, 2CNqA-2, pA-1^TA^, pA-1^TG^, pA-2), or 500 nm (Cy3). Emission was collected at a right angle with 1.0 nm wavelength interval (integration time: 0.05–0.1 s, optical path length: 3.0 mm) and monochromator slits adjusted to achieve optimal signal output, resulting in SBWs in the interval 1.0–2.8 nm on both the excitation and emission side. Emission spectra were corrected for Raman scattering by subtracting the corresponding emission from the solvent only.

#### Fluorescence quantum yield determinations

Fluorescence quantum yield (Φ_F_) determinations were performed using a duplex- or single-strand concentration of 9.5 µM (± 0.5 µM) and 60–100 µL sample volume in a 3.0 mm × 3.0 mm path length quartz cuvette. For the duplex samples (the RNA-bound gapmers), a 30–40% molar excess of RNA was applied to promote full hybridization. For the FBA-labeled gapmers, Φ_F_ was determined relative to a solution of quinine sulphate (Sigma) in 0.5 M H_2_SO_4_ (Φ_F,REF_ = 55%^50^). For the Cy3-labeled gapmer, a solution of Rhodamine 6G (Sigma) in ethanol (Φ_F,REF_ = 95%^50^) was used. Quantum yields were calculated according to:

$${\Phi }_{F}={\Phi }_{F,REF}\times \frac{\int {I}_{S}\left(\lambda \right)d\lambda }{\int {I}_{REF}\left(\lambda \right)d\lambda }\times \frac{{A}_{REF}}{{A}_{s}}\times \frac{{\eta }_{S}^{2}}{{\eta }_{REF}^{2}}$$

where I_S_(λ) and I_REF_(λ) denote the emission intensity of the sample and reference, respectively. The absorption at the excitation wavelength for the samples (A_S_) and reference (A_REF_) under the applied conditions were in the interval 0.004–0.05, to ensure negligible inner filter effects. The adopted solvent refractive index for the sample was η_S_ = 1.333 (phosphate buffer), while the corresponding values for the references were η_REF_ = 1.339 (for 0.5 M H_2_SO_4_) and η_REF_ = 1.360 (for ethanol). The reported quantum yields are given as mean ± standard deviation of two independent replicates.

#### Fluorescence lifetime measurements

Fluorescence lifetimes were determined with time-correlated single photon counting using identical sample conditions as in the quantum yield determination (vide supra). The FBA-labeled gapmers were excited using an LDH-P–C-375 (PicoQuant) pulsed laser diode with emission centered at 377 nm; full width half max (FWHM) of the pulse were *ca.* 1 nm and 70 ps with respect to wavelength and time, respectively. The Cy3-labeled gapmer was excited using an LDH-D-TA-560 (PicoQuant) pulsed laser diode with emission centered at 560 nm; FWHM of the pulse were *ca.* 0.5 nm and 40 ps with respect to wavelength and time, respectively. The laser diodes were powered by a PDL 800-B (PicoQuant) laser driver delivering light pulses at a frequency of 10 MHz. Sample emission was collected at a right angle, through an emission polarizer set at 54.9° (magic angle detection) and observed at λ_max_ of the sample emission (± 10 nm) with a 10 nm SBW. Photon counts were recorded on a R3809U-50 microchannel plate PMT (Hamamatsu) and fed into a LifeSpec multichannel analyzer (Edinburgh Analytical Instruments) with 1024 or 2048 channels (at 20–50 ps/channel resolution) until the stop condition of 10^4^ counts in the top channel was met. The instrument response function (IRF) was determined using a frosted glass (scattering) insert. The reported amplitude-averaged lifetimes are given as mean ± standard deviation of two independent replicates. Details on data fitting and calculation of average lifetimes are provided in Supplementary Sect. 2.4 and Table S1.

#### Absorption monitored gapmer:RNA duplex melting

Melting of the gapmer:RNA duplexes was performed at 4 µM duplex concentration using equimolar amounts (± 10%) of gapmer and RNA at a sample volume of 1 mL (± 10%). The melting was performed using a Cary 4000 or Cary 5000 (Varian Technologies) UV–vis spectrophotometer equipped with a Peltier unit for temperature control, by ramping the temperature from 20 °C to 85 °C at a rate of 1.0 °C/min, then holding at 85 °C for 5.0 min, then returning to 20 °C at a rate of 1.0 °C/min. This cycle was repeated twice (*i.e.* four melting transitions). Absorption at 260 nm was collected at 1.0 °C intervals (SBW: 2 nm, integration time: 2.0 s, optical path length: 4.0 mm) throughout the temperature program. After processing the absorption (A) *vs.* temperature (T) data with a five-point Savitzky-Golay smoothing function, the melting temperature (T_m_) was calculated as the mean ± standard deviation of the zero-intercepts of the second derivative (d^2^A/dT^2^ = 0) for the four melting transitions.

#### Circular dichroism (CD)

**Figure 2:**
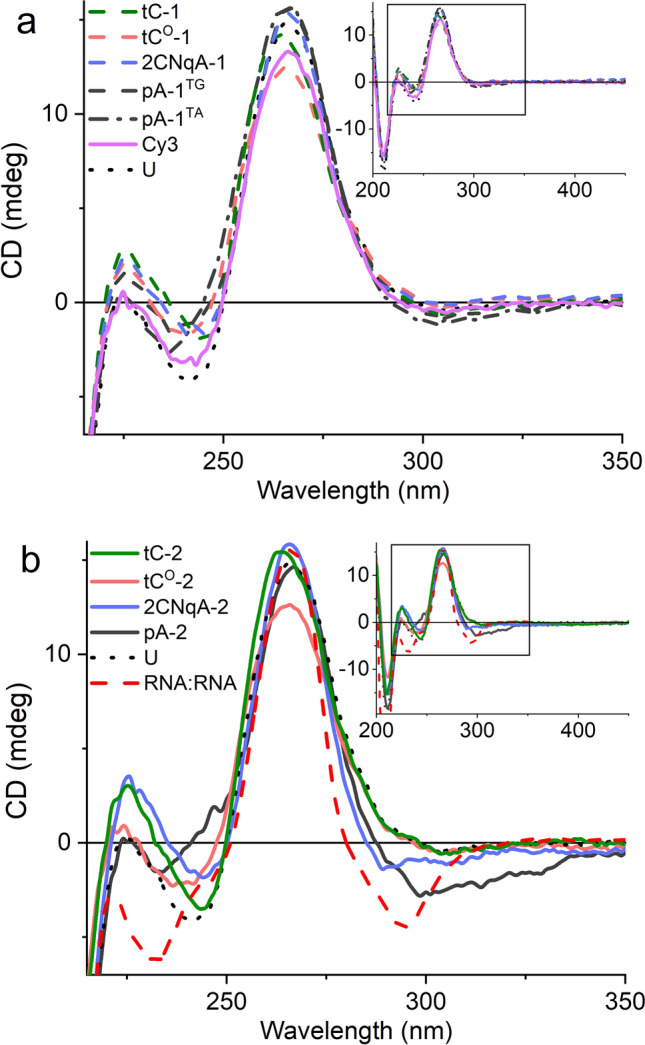
Circular dichroism (CD) spectra of tC (green), tC^O^ (red), 2CNqA (blue), pA (dark gray), Cy3 (violet), and unmodified U (black dotted line) gapmers hybridized to the RNA target T. The duplex concentration was 4 µM and the spectra were collected at 10–15 °C to ensure full hybridization. Insets: A zoom-out indicating the region (black rectangle) of the main figure. (**a**) Single-labeled gapmers. (**b**) Double-labeled gapmers. To illustrate the similarity to the A-form CD signature, an RNA:RNA duplex (red dashed line, normalized, see Material and Methods section for sequence) is included.

The CD spectra and CD-monitored melting of gapmer:RNA duplexes were performed using identical sample conditions as in the absorption melting (vide supra), on a Chirascan CD spectrometer (Applied Photophysics) equipped with a Peltier unit for temperature control. The spectra were recorded with a 1.0 nm wavelength interval (integration time: 0.3 s, optical path length: 4.0 mm, SBW: 1 nm) in two sequential repeats. The repeats were then averaged, and the corresponding spectrum of the solvent was subtracted. The temperature cycling was performed between 10 °C and 80 °C in steps of 5 °C or 10 °C, with a 5 min hold time at each temperature to allow for equilibrium to set. The CD melting temperature was determined as the inflection point of the Boltzmann sigmoid fits of the CD *vs.* T data (Supplementary Fig. S20) and presented as the mean ± standard deviation of the T_m_ for the two melting transitions. For the A-form CD spectrum presented in Fig. 2b, the following RNA sequence (hybridized to its complimentary RNA strand) was used: 5’-CGACACACACAAGGACGAGGAUUCC-3’.

### Real-time polymerase chain reaction (qPCR) for *MALAT1* knockdown

#### Cell culture

HEK 293 cells stably overexpressing glucagon-like peptide 1 receptor (HEK 293-GLP1R) were cultured in Dulbecco’s modified eagle medium (Gibco, 31,966) containing 10% fetal bovine serum (Gibco, 10,270) and 100 µg/ml hygromycin B (Invitrogen, 10,687) and were maintained at 37 °C in a humidified atmosphere containing 5% CO_2_, 21% O_2_.

#### Cell lysis, reverse transcription, and real-time polymerase chain reaction

For knockdown analysis of the gapmers, 384-well plates coated with Poly-D-Lysin (Corning, 354,663) were seeded with 7,000 HEK 293-GLP1R cells in 35 µL per well. Twenty-four hours later, the cells were treated with gapmers at indicated concentrations (14 points, 1:3 dilution dose–response for each gapmer) in duplicate. Twenty-four hours later, the gapmer-containing medium was removed from the wells, and the cells were washed once with Dulbecco’s phosphate buffer saline (Gibco,14,040). Cell lysis was performed by adding 30 µL lysis buffer composed of RLN lysis buffer (Qiagen, 74,182) and 4% RNAsecure RNase Inactivation Reagent (ThermoFisher, AM7006) to the wells. Lysates were transferred from the cell plate to a PCR plate (Axygen, PCR-384-RGD-C) containing Cells-to-CT Reagents for reverse transcription reaction (ThermoFisher, 4391852C), making the lysate 10% of the total volume. The resulting cDNA samples were then diluted 1:16 in master mix solutions containing hydrolysis probes for *MALAT1* (Applied Biosystems, Hs00273907_s1) or *HPRT1* (Applied Biosystems, Hs02800695_m1). Real-time PCR reactions were run on QuantStudio 7 Flex Real-Time PCR System (Applied Biosystems). Further details on how the reverse transcription reaction and real-time PCR reaction were performed can be found in Stulz *et al*^51^. The relative mRNA expression levels for *MALAT1* were calculated by normalizing the *MALAT1* expression levels to those of the reference gene *HPRT1,* (${2}^{-\Delta {C}_{q}}$, where ΔC_q_ is the difference in expression). The relative expression values were then normalized to the corresponding values from the negative control (untreated cells), for each gapmer. The dose–response fitting procedure is described in supplementary Sect. 3.3.

### Fluorescence microscopy, flow cytometry, and cytotoxicity

#### Cell culture

Wild-type HEK 293T cells were purchased from ATCC® and cultured at 37 °C and 5% CO_2_ in complete medium composed of DMEM GlutaMax low-glucose (Gibco, 21,885,025), with an addition of 10% fetal bovine serum (Gibco, 10,270,106). During dissociation/sub-cultivation, the cells were washed with DPBS calcium/magnesium free (Gibco, 14,190,250) and exposed to Trypsin-0.25% EDTA. Cells were tested and confirmed to be mycoplasma free.

#### Fluorescence microscopy

The cells were seeded in a CELLview™ glass-bottom quartering cell culture dishes (Greiner Bio-One, 627,870) at a density of 0.18 million cells/mL with 250 µL per compartment one day prior exposure. Cells were exposed to the gapmers for 24 h, at a final concentration of 3 µM in cell culture medium. Confocal images were acquired on live cells using an inverted Nikon C2 + confocal microscope equipped with a C2-DUVB GaAsP Detector Unit with variable emission bandpass, and an oil-immersion 60 × 1.4 Nikon APO objective. (Nikon Instruments, Amsterdam, Netherlands). The fluorescence was detected between 407–700 nm for the FBAs (following excitation with the 405 nm laser line) and 563–700 nm for Cy3 (exc. 561 nm), with the pinhole aperture was set at 1 Airy unit. All images were equally processed using ImageJ 1.53c^52^ and the corresponding unadjusted images are provided in Supplementary Fig. S27.

#### Flow cytometry

Cells were seeded in flat-bottom 96-well plates (VWR, 734–2327) at a density of 0.18 million cells/mL with 100 µL/well one day prior to exposure. Cells were exposed to gapmers at doses up to 3 µM (6 points: 1:3 dilution steps). Twenty-four hours after treatment the supernatants were collected and cells were washed with DPBS, dissociated using TrypLE™ Express (12,604,021), and resuspended in DPBS with 5% FBS. Cell samples were transferred to round bottom 96-well plates and analyzed on a BD LSRFortessa™ flow cytometer (BD Instruments). Debris were excluded from the analysis by plotting the forward scatter (FSC) *vs.* side scatter (SSC) with all events and gating for living cells. Cell aggregates (dividing cells and cell clusters) were excluded by plotting the SSC-height *vs.* SSC-area with living cells and gating for single cells. Cells were excited with 405 nm laser light, where after intracellular FBA mean fluorescence intensities were detected and treated as described in supplementary Sect. 3.5. Experiments were performed in biological duplicates with three technical replicates per biological replicate.

#### Cytotoxicity

Cell membrane integrity was evaluated using the CyQUANT™ LDH Cytotoxicity Assay (Thermo Scientific, C20300) according to the manufacturer’s instructions. Briefly, reduced lactate dehydrogenase (LDH) released in the supernatants of cells exposed to gapmers for 24 h was measured using a coupled enzymatic assay which results in the conversion of a tetrazolium salt into a red formazan product. The product absorption was recorded at 490 nm and 680 nm. The toxicity was expressed as the percentage of LDH release in supernatant compared to maximum LDH release (supernatant + cell lysate) and the normalized fraction of cell death was calculated by dividing the LDH values by the corresponding values from lysed untreated cells.

## Results and discussion

### Target affinity, knockdown efficiency, and secondary structure of the gapmers

#### Target affinity and MALAT1 knockdown

We opted for using tC to explore the impact of different labeling positions in the gapmer, due to its robust fluorescence and cytosine-mimicking properties reported in a variety of nucleic acid surroundings^41,42^. We synthesized four sequences with different tC substitution patterns (tC-1, tC-2, tC-2^w^, and tC-3, Table 1) and tested their affinity to the fully complementary RNA target sequence (T, Table 1) using UV-monitored melting experiments as well as *MALAT1* knockdown efficiency in HEK 293-GLP1R cells using a direct lysis real-time polymerase chain reaction (qPCR) assay.

**Table 1:** Gapmer sequences, gapmer:RNA melting temperatures, and knockdown efficiencies, relative to their unmodified counterpart U.

Oligomer	5’–3’ Sequence (X = FBA^*a*^)	Fluorescent label	ΔT_m_ (°C)^*b*^	ΔpEC_50_ (log(M))^*c*^
U	**GCA**TTCTAATAGC**AGC**	*–*	0 ± 0.5	0 ± 0.3
U^w^	**G**C**A**TTCTAATAGC**AGC**^*d*^	*–*	− 1.7 ± 0.5	− 0.01 ± 0.3
tC-1	**GCA**TTCTAATAGX**AGC**	tC	− 1.8 ± 0.5	0.2 ± 0.4
tC-2	**GCA**TTXTAATAGX**AGC**	tC	− 0.8 ± 0.7	0.3 ± 0.4
tC-2^w^	**G**X**A**TTCTAATAGX**AGC**	tC	− 5.3 ± 0.8	0.3 ± 0.4
tC-3	**G**X**A**TTXTAATAGX**AGC**	tC	− 3.7 ± 0.5	0.3 ± 0.4
tC^O^-1	**GCA**TTCTAATAGX**AGC**	tC^O^	− 1.6 ± 0.4	0.2 ± 0.4
tC^O^-2	**GCA**TTXTAATAGX**AGC**	tC^O^	0.9 ± 0.4	0.1 ± 0.4
2CNqA-1	**GCA**TTCTAATXGC**AGC**	2CNqA	2.0 ± 0.4	0.1 ± 0.3
2CNqA-2	**GCA**TTCTXATXGC**AGC**	2CNqA	4.0 ± 0.4	0.6 ± 0.4
pA-1^TG^	**GCA**TTCTAATXGC**AGC**	pA	− 0.9 ± 0.4	0.6 ± 1.5^*e*^
pA-1^TA^	**GCA**TTCTXATAGC**AGC**	pA	3.6 ± 0.4	0.5 ± 0.3^*e*^
pA-2	**GCA**TTCTXATXGC**AGC**	pA	− 0.1 ± 0.4	–^*f*^
Cy3	Cy3-TCA**GCA**TTCTAATAGC**AGC**^*g*^	Cy3	0.5 ± 0.4	0.4 ± 0.7^* h*^
T	GCUGCUAUUAGAAUGC^*i*^	*–*	*n.a*	*n.a*

Bold font denotes cEt sugars; all gapmers have phosphorothioate backbones throughout the sequence.

^*a*^The sugar moiety in the FBA nucleotides is deoxyribose.

^*b*^Difference in melting temperature compared to the unmodified gapmer U (T_m_(U) = 61.5 ± 0.4 °C) in 10 mM phosphate buffer (pH 7.4) with 100 mM NaCl and 1.0 mM EDTA added, mean ± standard deviation of four melting transitions.

^*c*^Difference in effective *MALAT1* knockdown concentration compared to the unmodified gapmer U (pEC_50_(U) = − 5.7 ± 0.2, EC_50_ unit: M), mean ± standard deviation of duplicate biological replicates run on at least two occasions.

^*d*^Gapmer U^w^, having one deoxyribose sugar in position 2, was included as a control for the wing-labeled FBA-gapmers tC-2^w^ and tC-3.

^*e*^Unsatisfactory fit due to incomplete knockdown in the applied concentration range, see Supplementary Fig. S22.

^*f*^Unable to fit data.

^*g*^Cy3 was conjugated to the 5’ end of the gapmer via a DBCO-type click linker (Supplementary Fig. S14).

^*h*^The Cy3 gapmer was evaluated in a biological duplicate on one occasion and the pEC_50_ was referenced to the unmodified gapmer U included in the same experiment (Supplementary Fig. S23).

^*i*^Target sequence, all RNA.

The melting temperature (T_m_) of the unmodified gapmer (U, Table 1) with its complementary RNA T was determined to be 61.5 ± 0.4 °C and, like all gapmers included in this study, it displays a distinct two-state melting behavior (Supplementary Fig. S21 and Table S3). It has previously been shown that the effect on T_m_ of substituting canonical bases for FBAs typically depends on the neighboring bases in the oligonucleotide; for example, tC substitution in 10mer DNA renders a ΔT_m_ of 5 °C for TT neighbors, but − 1 °C for GA neighbours^42^, which are the positions for the cytosine analogues in this work. Here, we observe a small but consistent destabilizing effect on the gapmer:RNA duplex for all combinations of tC incorporations in the gapmers, with ΔT_m_ ranging from − 0.8 to − 5.3 °C (Table 1). Because the FBA phosphoramidites we used to synthesize the gapmers have deoxyribose sugars instead of an affinity-promoting cEt^48^, the introduction of a tC in the wing, like for tC-3 and tC-2^w^, effectively replaces one cEt sugar with a deoxyribose in the gapmer. To isolate this feature, we synthesized the control gapmer U^w^, which is identical to the unmodified gapmer U apart from having a deoxyribose in position 2. The observed negative ΔT_m_ for U^w^ (− 1.7 °C) suggests that the reduced affinity for tC-3 and tC-2^w^ in part, but not fully, can be attributed to the removal of a cEt sugar. We thereafter tested the knockdown efficiency of the tC-containing gapmers. The unmodified gapmer U has a pEC_50_ of − 5.7 ± 0.2 (Table 1, see Supplementary Figs. S22–S23 and Table S4 for dose–response data; unit for EC_50_: M), and we found that tC modifications barely affect the gapmer activity (ΔpEC_50_ = 0.2–0.3), with no apparent differences between tCs positioned in the wings and gap of the sequence.

We next explored the other types of FBAs and decided to challenge our in-sequence labeling approach by incorporating them in the biologically critical gap region, rather than in the wing of the gapmer. This led to the synthesis of gapmers containing one or two incorporations of tC^O^, 2CNqA, or pA in the gap, as shown in Table 1. The ΔT_m_ observed for the gap-labeled sequences is consistently small; the most extreme effect being an increase of *ca.* 4 °C, as seen for 2CNqA-2 and pA-1^TA^, which is in line with the corresponding behavior reported for 2CNqA and pA in 10mer DNA^34,35^. The knockdown efficiency of the FBA gapmers were similar to that of the unmodified gapmer for sequences containing tC^O^ (ΔpEC_50_ = 0.1–0.2), while 2CNqA-2 and the pA-containing gapmers exhibited a reduced activity (ΔpEC_50_ = 0.5–0.6).This was especially apparent for pA-2, for which a full response curve could not be captured in the assayed dose range (Supplementary Fig. S22). Although gapmer-induced gene knockdown involves several interlaced processes, including uptake, RNA binding, and RNase H1 processing, it is tempting to ascribe the reduced activity of in particular 2CNqA-2 and pA-2 to their fairly sizeable quadra- and pentacyclic ring systems, respectively, in combination with the proximity of the FBA moieties in the gap. For comparison, we included a Cy3 end-labeled gapmer in this study (Cy3, Table 1). We note that the presence of the fluorophore in this position has a negligible effect on the gapmer:RNA duplex stability (ΔT_m_ = 0.5 °C) but that it reduces the knockdown efficiency slightly (ΔpEC_50_ = 0.4).

#### Secondary structure of gapmer:RNA duplexes

To investigate the effect of FBA incorporation on gapmer:RNA secondary structure, we measured circular dichroism (CD) of the gapmers hybridized to the RNA target sequence T.

The resulting CD spectra (Fig. 2) are dominated by strong positive bands at *ca.* 265 nm and weak negative bands at *ca.* 240 nm, which closely resembles the features of locked nucleic acid:RNA duplexes^53,54^. We also note that the spectra share common features with the RNA homoduplex spectrum, most notably the strong band at 265 nm (Fig. 2b), which agrees with the reported notion that RNA forms duplexes that are predominantly A-form when hybridized with a DNA strand containing locked nucleotides^55^. Induced CD, which may arise as a result of incorporating FBAs into nucleic acid structures, was not observed, as evidence by the lack of spectral features in the 330–420 nm region (Fig. 2, insets). We conclude that all single-labeled gapmers, including Cy3, give rise to CD spectra that closely resemble that of the unmodified gapmer U (Fig. 2a), which suggests that the fluorophores do not perturb the secondary structure of the hybrid duplex. This is also true for the double-labeled gapmers, with the exceptions of 2CNqA-2 and in particular pA-2, which both have slightly deviating CD signatures in the 280–340 nm region (Fig. 2b). This is interesting in relation to their reduced knockdown efficiency (vide supra), suggesting that the interactions between the RNase H1 and gapmer:RNA duplexes could be compromised when the two adenine FBAs are positioned close to each other in the gap. In addition to the CD spectra shown in Fig. 2, the temperature dependence of the CD for all gapmers are provided in Supplementary Fig. S20 and Table S2. As expected, we observe the same two-state hybridization behavior as in the corresponding isotropic absorption melting experiment, and the T_m_ data extracted from the CD support the trend seen in the UV absorption data.

### Photophysical properties of the labeled gapmers

Understanding the photophysical properties of a fluorophore is essential for accurate interpretation of the behavior of the labeled oligonucleotide and for assessing compatibility with other fluorescence- and microscopy-based applications. To facilitate this, we conducted a thorough assessment of the modified gapmers, examining their spectral characteristics and fluorescence quantum yields (Φ_F_) using UV–vis- and fluorescence spectroscopy, as well as fluorescence lifetimes (τ_F_) using time-correlated single photon counting (TCSPC).

**Figure 3:**
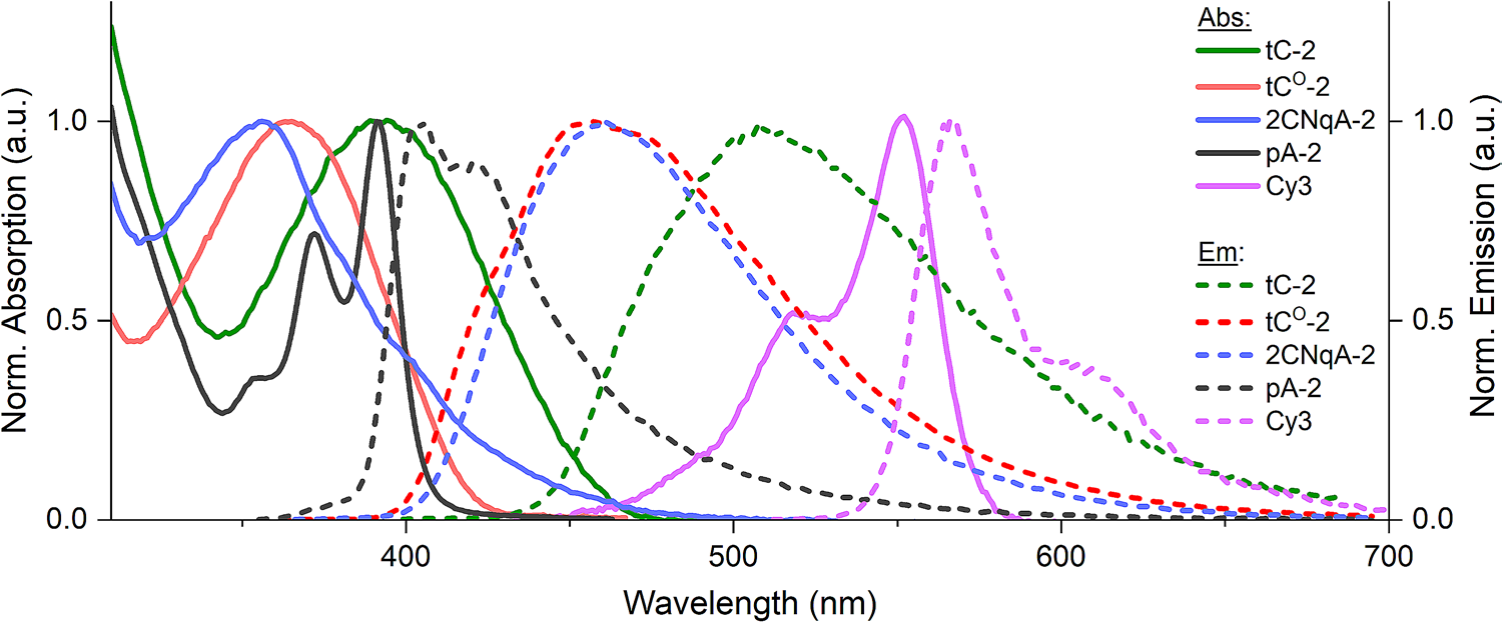
Normalized UV–vis absorption spectra (solid lines) and emission spectra (dashed lines) of the tC-2 (green), tC^O^-2 (red), 2CNqA-2 (blue), pA-2 (black), and Cy3 (violet) gapmers.

**Figure 4:**
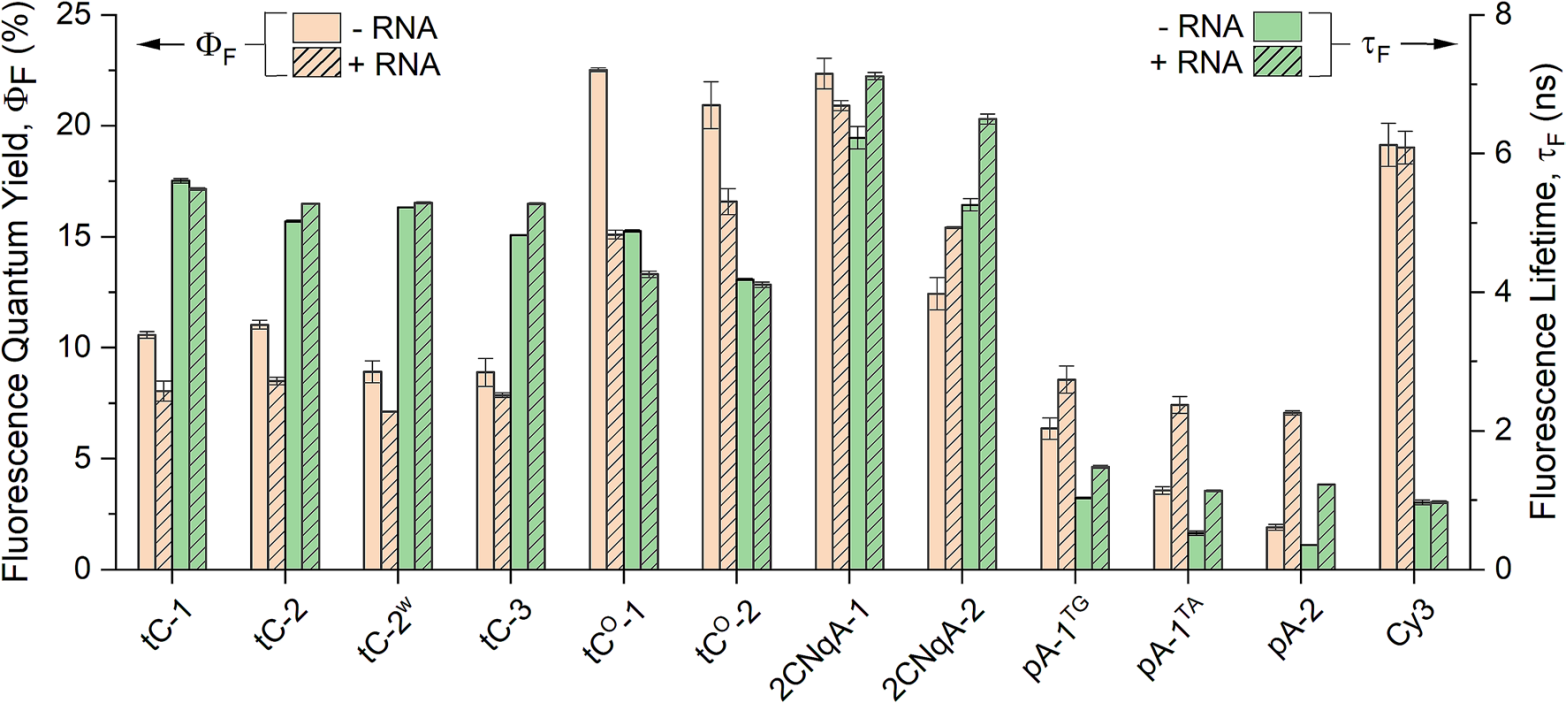
Fluorescence quantum yield (Φ_F_, red bars) and lifetime (τ_F_, green bars) of the single-stranded (plain bars) and RNA-bound (striped bars) gapmers. Presented values are mean ± standard deviation from two independent replicates. The corresponding numeric data are provided in Supplementary Table S1.

*Single-stranded gapmers*. The absorption spectra of the FBA-labeled gapmers are characterized by bands centered at *ca.* 260 nm, corresponding to the absorption of the canonical nucleobases, and FBA bands in the 320–470 nm region, as exemplified in Fig. 3. Upon excitation into the FBA band the tC-, tC^O^-, and 2CNqA-labeled gapmers display a broad unstructured emission in the 400–600 nm region, while the pA gapmers differ by having split emission bands and significantly smaller Stokes shifts. The complete set of absorption and emission spectra of all gapmers, both single-stranded and RNA-bound, are provided in Supplementary Fig. S19. Overall, both the absorption and emission features of the FBAs in the gapmers resemble those reported for the corresponding FBAs inside RNA and/or DNA^33–35,40,41^. The spectra of the Cy3 gapmer are strongly reminiscent of what has been reported for Cy3 previously in several different environments^56^. The gapmers’ emissive properties were further characterized by determining Φ_F_ and τ_F_ (Fig. 4).

The emissive properties of tC are known to be comparably insensitive to neighboring bases^41^, which is also what is observed here, with Φ_F_ and τ_F_ for the single-stranded tC gapmers ranging from 8.9%–11% and 4.8–5.6 ns, respectively (see Supplementary Fig. S19 and Table S1 for detailed fluorescence decay data). However, the Φ_F_ observed for tC in the gapmer is substantially lower than those reported in ssDNA (< Φ_F_ >  = 20%, < τ_F_ >  = 5.7 ns for mixed neighbours^41^), which is surprising considering that a corresponding change in τ_F_ is not observed. This behavior could be explained by ground state quenching occurring in parts of the fluorophore population, but we also note that Φ_F_ close to 10% has been suggested for tC in single-stranded RNA (ssRNA) before^57^. In contrast to tC, the emissive properties of tC^O^ in single-stranded oligonucleotides have been shown to vary substantially depending on neighboring bases^33^ and in DNA^33^ compared to RNA^40^. Here, we observe a Φ_F_ of 23% and 21% for tC^O^-1 and tC^O^-2, respectively. Although this is moderately lower than what has been reported in ssDNA (< Φ_F_ >  = 30% for mixed neighbours^33^), tC^O^-1 has the highest Φ_F_ noted in this work. The adenine analogues 2CNqA and pA both have thymines as neighboring bases in the herein investigated gapmer sequence (TG and/or TA neighbors), which is known to have a negative impact on Φ_F_ for these FBAs^34,35^. The 2CNqA-1 and 2CNqA-2 gapmers exhibit Φ_F_ of 22% and 12%, respectively, which is in fair agreement with the Φ_F_ of 14%–15% reported in ssDNA with the same neighbors. The pA-labeled gapmers have comparably poor Φ_F_ (1.9%–6.4%) although again consistent with previous reports for pA in ssDNA^35^. The observation that Φ_F_ for pA-2 is significantly lower than for pA-1^TG^ and pA-1^TA^ may indicate an interbase quenching interaction between the two FBA moieties in pA-2, which would agree with the findings of a slightly deviating duplex CD (vide supra). The Φ_F_ for the Cy3 gapmer in this work was determined to 19%, which is in the low end of what has been reported for Cy3-labeled short oligomers (18%–39% in ssDNA, depending on sequence)^58^. Lastly, we analyzed the brightness of the gapmers (Φ_F_ × molar absorptivity, Supplementary Fig. S18) and note that the highest ranking gapmer in this respect is tC^O^-2, having 3,400 M^-1^ cm^-1^ and 1,100 M^-1^ cm^-1^ at the FBA band maximum (367 nm for tC^O^-2) and at 405 nm, respectively. This is still significantly dimmer than Cy3 which, based on the Φ_F_ determined here, has a maximum brightness that is approximately 8 times higher.

*RNA-bound gapmers.* Studies aiming at quantifying uptake into cells and tissue by fluorescent means rely on fluorophores that maintain constant (or highly predictable) emissive properties, regardless of hybridization status, to report emission that is proportional to ASO concentration. If the goal instead is to study ASO-RNA interactions, a fluorophore arrangement that significantly increases in brightness, changes fluorescence lifetime, and/or displays a spectral shift upon duplex formation is preferred. To explore the FBA gapmers on these premises, we decided to investigate the photophysical properties after hybridization to form a heteroduplex with the fully complementary RNA. The Φ_F_ and τ_F_ for the gapmer:RNA duplexes are shown in Fig. 4 (striped bars), while absorption and emission spectra, as well as fluorescence decays are shown in Supplementary Fig. S19. We find that RNA-binding has a minor effect on the emission characteristics of the tC gapmers, which is in line with the corresponding reports on homoduplex formation in DNA^41^. For the tC^O^ gapmers, however, a small-to-moderate drop in Φ_F_ is observed (4–8 percentage points), which again agrees with the small hybridization-induced changes observed for tC^O^ in DNA and RNA homoduplexes^33,40^_._ For the 2CNqA gapmers, RNA binding brings about a small increase in Φ_F_ and τ_F_ for 2CNqA-2, while a notable blue-shift of 6–8 nm is observed in the emission spectra of both 2CNqA gapmers (Supplementary Fig. S19g–h). The 1.3-fold increase in Φ_F_ observed for 2CNqA-2 is, however, modest compared to the corresponding behavior of 2CNqA with one or two thymine neighbors in ssDNA, where Φ_F_ increases by a factor of *ca.* 2–3 upon forming a homoduplex^34^. The pA differs from the other FBAs in the gapmer environment by exhibiting a consistent increase in both Φ_F_ and τ_F_ upon binding to the target RNA, with Φ_F_ increasing by a factor of 1.3, 2.1, and 3.7 for pA-1^TG^, pA-1^TA^, and pA-2, respectively. A hybridization-induced increase in Φ_F_ and τ_F_ of comparable magnitude has been reported for DNA-incorporated pA with one or two thymine neighbors before^35^ and suggests that it may be possible to use fluorescence lifetime recording to detect target hybridization with pA-containing gapmer strands. The emissive properties of the Cy3 gapmer are virtually unchanged upon RNA binding, suggesting that the end-grafted Cy3 moiety does not interact with the base stack in this gapmer. In conclusion, we find representatives of two different types of labeling strategies among our gapmers, where for instance the tC and 2CNqA gapmers are fairly bright and insensitive to microenvironment, and thus suitable for fluorescence-based quantification, while pA-2, although having a low brightness as a single-strand, has the principal qualities of a hybridization probe, owing to its 3.7- and 3.4-fold increase in Φ_F_ and τ_F_, respectively, upon binding to RNA.

### Cellular uptake of labeled gapmers

To demonstrate the applicability of the FBA-labeled gapmers in a biological context, we studied their uptake and localization in human embryonic kidney cells (HEK 293 T) using fluorescence microscopy and flow cytometry. For these experiments, we focused on the gapmers labeled with one or two FBAs in the gap, and on comparisons to the Cy3 gapmer. All gapmers were non-toxic to the cells at concentrations up to 3 µM, which is the highest dose applied in the live cell experiments, as shown by lack of lactate dehydrogenase (LDH) leakage after 24 h of incubation at 37 °C (Supplementary Fig. S25).

#### Fluorescence microscopy

**Figure 5:**
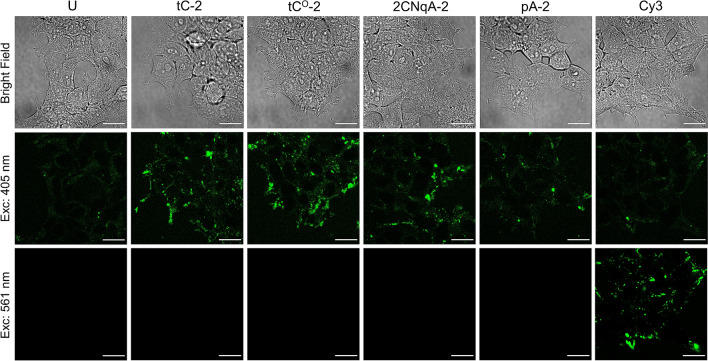
Confocal microscopy images of live HEK 293 T cells continuously exposed to unformulated gapmers (3 µM) for 24 h. Samples were excited at 405 nm (emission: 407–700 nm) for FBA detection and at 561 nm (emission: 563–700 nm) for Cy3 detection. Scale bars are 20 µm.

We exposed cells to 3 µM unformulated gapmers for 24 h at 37 °C, where after the cells were immediately transferred to a stage-top incubator and imaged using a confocal microscope. The acquired images (Fig. 5; see Supplementary Fig. S26 for the corresponding data for the gapmers labeled with one FBA) show that the gapmers are internalized and accumulate inside the cells.

**Figure 6:**
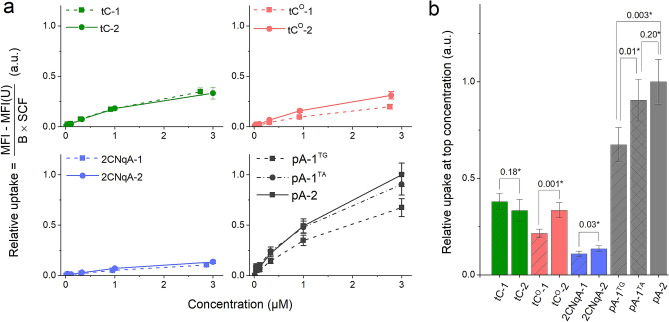
Quantification of gapmer uptake in HEK 293 T cells after 24 h continuous exposure, evaluated using flow cytometry. All data were collected using the same excitation power (excitation at 405 nm) and detector settings. To enable comparisons of the FBA-labeled gapmer concentrations inside cells, a relative uptake was calculated based on the observed mean fluorescence intensity (MFI, see Supplementary Fig. S24 for the untreated data). Briefly, this was achieved by (*1*) subtracting the corresponding MFI from the negative control (unmodified gapmer U at equal concentration), (*2*) dividing by the brightness of the single-stranded gapmer (B) and (*3*) dividing by a spectral correction factor (SCF), see supplementary Sect. 3.5 for details. (**a**) Dose-dependent relative uptake. (**b**) Relative uptake at the top concentration in (**a**). Error bars are propagated standard deviations (*N* = 6) based on the cytometry- and brightness data (Supplementary Fig. S18). *Probability for equal mean from a paired sample *t*-test.

The observed punctuate intracellular fluorescence suggests that the uptake occurs occurred via endocytosis, resulting in vesicular localization of the gapmers, which is consistent with the recognized uptake process for oligonucleotide gymnosis^59^. We furthermore note that the punctuate distribution of FBA-labeled gapmers is similar to that of the Cy3 gapmer, suggesting that no significant difference in uptake route exist under the conditions applied here. To quantify the uptake of the FBA-labeled gapmers, we proceeded to study the dose-dependence of the uptake using flow cytometry (Fig. 6).

To correlate the mean fluorescence intensity signal (MFI) emanating from the cells in the flow cytometry experiment to gapmer concentration, we take into account the brightness and emission spectra of each FBA gapmer according to the procedure described in supplementary Sect. 3.5. This allowed us to compare the relative accumulation of the FBA-containing gapmers in the cells, as shown in Fig. 6. It should be noted that the herein presented uptake is based on total intracellular fluorescence*, i.e.* it does not distinguish between intra- and extra-endosomal emission and therefore does not reflect functional (or *productive*) uptake. We observe that gapmer uptake increases with the applied dose, approaching saturation at the highest applied dose (3 µM, Fig. 6a). Judging by the difference in MFI of treated and untreated cells as well as the level of uncertainty in the cytometry data, it is clear that the brightness of the FBA-labeled gapmers is sufficient to reliably detect their uptake at an applied dose of 300 nM, despite the use of 405 nm excitation, which from a brightness perspective is suboptimal for all FBAs except tC (Supplementary Fig. S18). We furthermore observe that having two FBA incorporations, compared to one, leads to a small-to-moderate, albeit statistically significant (paired sample *t*-test, Fig. 6b), increase in uptake for tC^O^, 2CNqA, and pA-1^TG^, but not for tC and pA-1^TA^. This could be due to the slight increase in lipophilicity that result from the doubled aromatic moieties in the gapmer. The most striking feature of the flow cytometry data, however, is the distinctly higher uptake observed for the pA gapmers; in particular pA-2, which exhibit an uptake that is 3.0-fold higher than tC-2 and tC^O^-2, and 7.4-fold higher than 2CNqA-2. It is noteworthy that the gapmers with the most pronounced uptake also are the ones for which a substantial fluorescence turn-on effect was observed upon binding to RNA: 2.1- and 3.7-fold for pA-1^TA^ and pA-2, respectively (vide supra). However, considering the punctuate distribution of ASOs inside the cells, we find it unlikely that a significant fraction of the gapmer population has escaped the endosomes and bound to RNA in our experiments. Nevertheless, it cannot be excluded that a hybridization-induced increase in Φ_F_ to some extent contributes to an overestimated uptake for the pA gapmers. Studies on the pA monomer have revealed it to be relatively insensitive to solvent polarity: Φ_F_ = 66%, 84%, and 74% in water, dimethyl sulfoxide, and toluene, respectively^35^, which further suggests that the observed MFIs for the pA gapmers are unlikely to be skewed by environment-dependent effects, and rather reflect a difference in cell uptake. Finally, it should be noted that the complex relationship between total- and functional uptake of ASOs, which is intermediated by the highly elusive process of endosomal escape, is not fully understood^60,61^. For instance, Linanne et al. showed in a recent study that cEt-modified gapmer gymnosis differs significantly across cancer cell lines and suggested that knockdown activity does not correlate with internalized concentration^62^. Our observations are consistent with this notion since pA gapmers appear to have the highest uptake while also standing out from the rest of the FBA gapmers by exhibiting reduced knockdown activity. However, since neither endosomal escape nor RNase H1 processability are explicitly studied in this work, it is difficult to assess in which proportion the observed effect on *MALAT1* knockdown can be attributed to differences in total uptake, gapmer:RNA secondary structure, and RNA affinity.

## Conclusions

In this work, we synthesized modified versions of a 16 nt *MALAT1*-targeting gapmer containing the fluorescent base analogues (FBAs) tC, tC^O^, 2CNqA, and pA, and systematically investigated the impact of FBA incorporation on RNA binding affinity, gapmer:RNA secondary structure, photophysical properties, and knockdown efficiency. To demonstrate the applicability of the FBA labeling approach, we also used the gapmers to study gymnotic uptake into human cells by means of live cell fluorescence microscopy and flow cytometry. We report that incorporation of one or two FBAs in the gap region of the gapmer does not lead to substantial changes in RNA affinity for any of the investigated FBAs. Moreover, the gapmer:RNA secondary structure is unaffected by the presence of the FBAs, except for slight deviations for 2CNqA-2 and pA-2. We furthermore demonstrate that FBA-labeling is a viable alternative to external dyes for live cell studies of gapmer uptake and that all investigated FBAs, despite exhibiting significantly lower brightness than commercial dyes, can readily be detected with a conventional confocal microscope. This is further highlighted by the results in flow cytometry, where administered gapmer concentrations down to at least 300 nM were clearly detectable inside the cells, even for the low-brightness pA-2. We also report a distinctly higher uptake of gapmers containing pA compared to the other FBAs, suggesting that pA may be interesting to explore further as a building block for enhancing ASO uptake. Finally, the qPCR data show that, except for the pA gapmers and the doubly labeled 2CNqA-2, FBA incorporations do not perturb the knockdown efficiency. This importantly demonstrate that the FBAs can be used in the biologically critical gap-region and that tC, tC^O^, and 2CNqA in particular, are excellent mimics of their corresponding canonical bases in the gapmer environment. We foresee that FBAs will be further applied, not only as fluorescent probes for localization, but also in more advanced structural and mechanistic investigations involving ASOs and siRNA.

## Supplementary Information

Supplementary Information.

## Data Availability

The flow cytometry data are available at FlowRepository under repository ID FR-FCM-Z38. All other datasets generated and/or analyzed during the current study are available from the corresponding author on reasonable request.
