# Supplementary material for: Fluorescent base analogues in gapmers enable stealth labeling of antisense oligonucleotide therapeutics
Source: Sci Rep. 2021 May 31;11:11365. doi: 10.1038/s41598-021-90629-1 (PMC8166847; doi:10.1038/s41598-021-90629-1)
Supplement: Supplementary file 1 — Supplementary Information. [file 41598_2021_90629_MOESM1_ESM.pdf]

Supplementary Information for:

Fluorescent base analogues in gapmers enable stealth labeling of antisense oligonucleotide therapeutics

Jesper R. Nilsson,[1] Tom Baladi,[1,2] Audrey Gallud,[3] Dženita Baždarević,[4] Malin Lemurell,[2] Elin K. Esbjörner,[3] L. Marcus Wilhelmsson,[1] Anders Dahlén[5,*]

[1] Department of Chemistry and Chemical Engineering, Chalmers University of Technology, Gothenburg SE-412 96, Sweden.
[2] Medicinal Chemistry, Research and Early Development, Cardiovascular, Renal and Metabolism (CVRM), BioPharmaceuticals R&D, AstraZeneca, Gothenburg, Sweden.
[3] Department of Biology and Biological Engineering, Chalmers University of Technology, Gothenburg SE-41296, Sweden.
[4] Bioscience, Research and Early Development, Cardiovascular, Renal and Metabolism (CVRM), BioPharmaceuticals R&D, AstraZeneca, Gothenburg, Sweden.
[5] Oligonucleotide Discovery, Discovery Sciences, R&D, AstraZeneca, Gothenburg, Sweden.

* To whom correspondence should be addressed. Anders Dahlén, Tel: +46-31-706 4842, Email: anders.dahlen@astrazeneca.com

Present Address: Tom Baladi, Oligonucleotide Discovery, Discovery Sciences, BioPharmaceuticals R&D, AstraZeneca, Gothenburg, Sweden. Audrey Gallud, Advanced Drug Delivery, Pharmaceutical Sciences, R&D, AstraZeneca, Gothenburg, Sweden.

Content

ENTRY **PAGE**

1 Synthesis ... 2
  1.1 HPLC-MS and HRMS ... 2
  1.2 Cy3-labeling of gapmers ... 15
2 Spectroscopic characterization ... 17
  2.1 Molar absorptivity spectra of FBA-labeled gapmers ... 17
  2.2 Brightness of FBA-labeled gapmers ... 17
  2.3 Absorption, emission, and fluorescence decay data in the absence and presence of RNA ... 18
  2.4 Fitting of fluorescence lifetimes ... 22
  2.5 Circular dichroism of gapmer:RNA duplexes ... 23
  2.6 UV-monitored melting of gapmer:RNA duplexes ... 26
3 Biological evaluation ... 29
  3.1 *MALAT1* knockdown efficiency of the FBA-labeled gapmers ... 29
  3.2 *MALAT1* knockdown efficiency of the Cy3-labeled gapmer ... 30
  3.3 Fitting of dose-response curves ... 30
  3.4 Flow cytometry ... 31
  3.5 Adjustment for filter spectra and emission spectrum in the flow cytometry ... 31
  3.6 Cytotoxicity ... 32
  3.7 Confocal microscopy ... 33

1 SYNTHESIS

1.1 HPLC-MS and HRMS

HPLC-MS Methods:

A: Purification on XBridge C_{18}, 5 µm 19×150 mm column using a 20 mL/min gradient from 5 to 20 (10 min) then 20% to 30% (8 min) ACN in 0.1 M DIPEA acetate (pH 6.8) at 20 °C. Analysis on Waters Acquity BEH C_{18}, 1.7 µm 2.1×100 mm column using a 0.6 mL/min gradient from 20% to 70% acetonitrile in 10 mM tributylammonium acetate in 8 min at 60 °C.

B: Purification on a Clarity Oligo RP, 5 µm 21.2×150 mm column using a 25 ml/min gradient from 10% to 70% (10 min) then 70% to 90% (3 min) MeOH in 50 mM ammonium carbonate (pH 8) at 20 °C. Analysis on a Clarity Oligo RP, 3 µm 4.6×150 mm column using a 0.6 ml/min gradient from 10% to 90% MeOH in 50 mM ammonium carbonate (pH 8) in 10 min at 45 °C.

C: Purification on Clarity Oligo RP, 5 µm 21.2×150 mm column using a 25 ml/min gradient from 10% to 50% (10 min) then 50% to 90% (4 min) ACN in 50 mM ammonium carbonate (pH 8) at RT. Analysis on Clarity Oligo RP, 3 µm 4.6×150 mm column by using a 0.6 ml/min gradient from 10% to 90% acetonitrile in 50 mM ammonium carbonate (pH 8) in 10 min at 45 °C.

HRMS Method:

Elution using a 0.3 ml/min gradient from 20% to 70% (8 min) ACN in 10 mM tributylammonium acetate (pH 10) at 60 °C on Waters Bioaccord system with Tof mass analyzer. Ionization mode: ESI negative. Source capillary voltage: 800 V. Source desolvation temperature: 550 °C. Mass range: 400–5,000 Da.

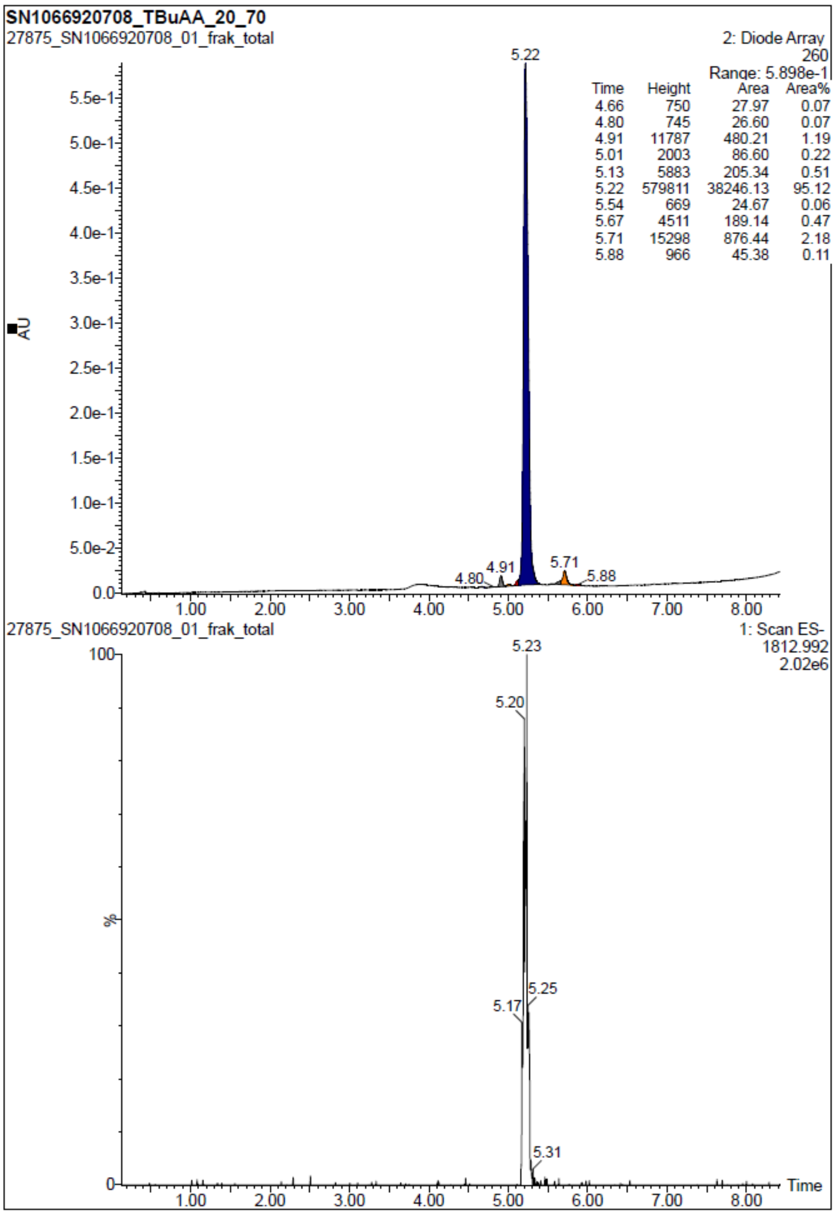

Figure S1. HPLC-MS and HRMS for gapmer U, obtained as a white powder using HPLC method A (NH_3 salt, 43%); UV purity 95%; *m/z* calcd 5410.6432; found: 5410.6037.

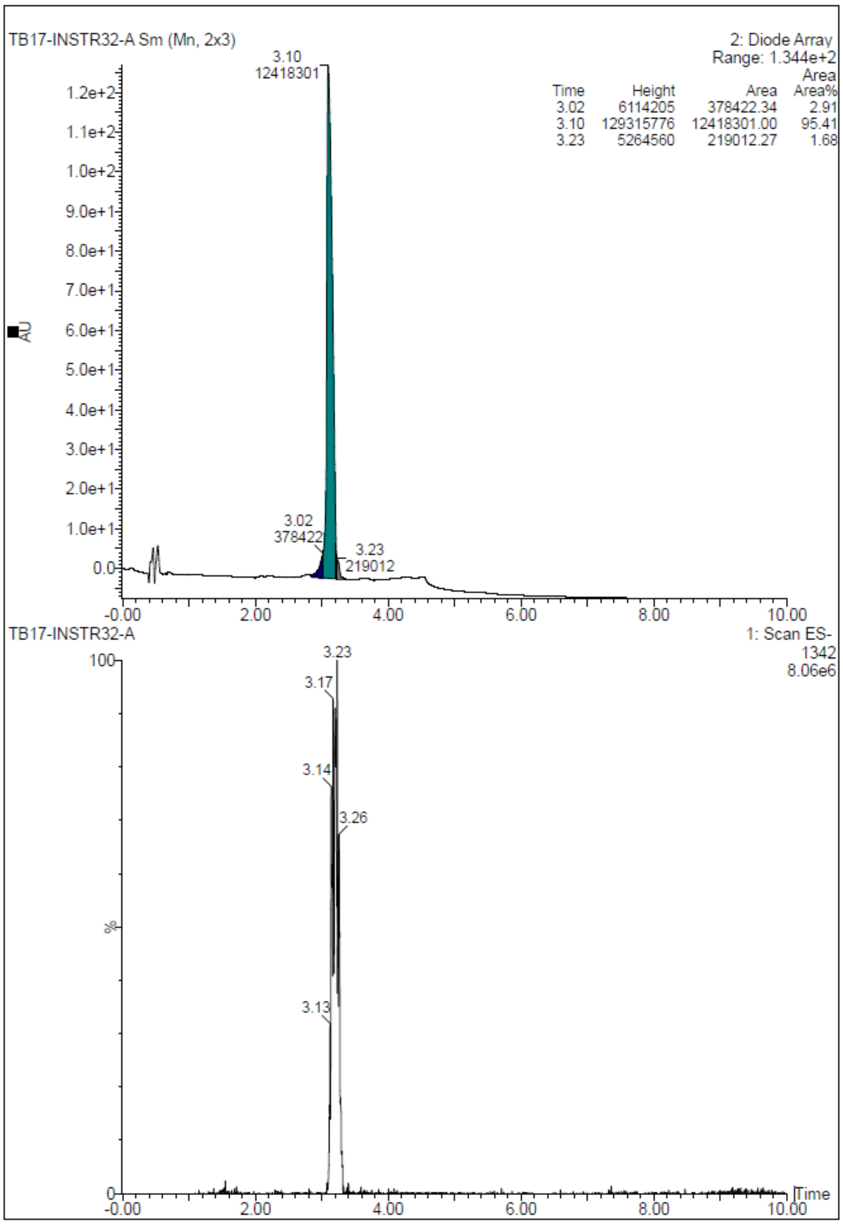

Figure S2. HPLC-MS and HRMS for gapmer U[w], obtained as a yellow powder using HPLC method A (NH_3 salt, 33%); UV purity 95%; *m*/*z* calcd 5368.6326; found: 5368.6377.

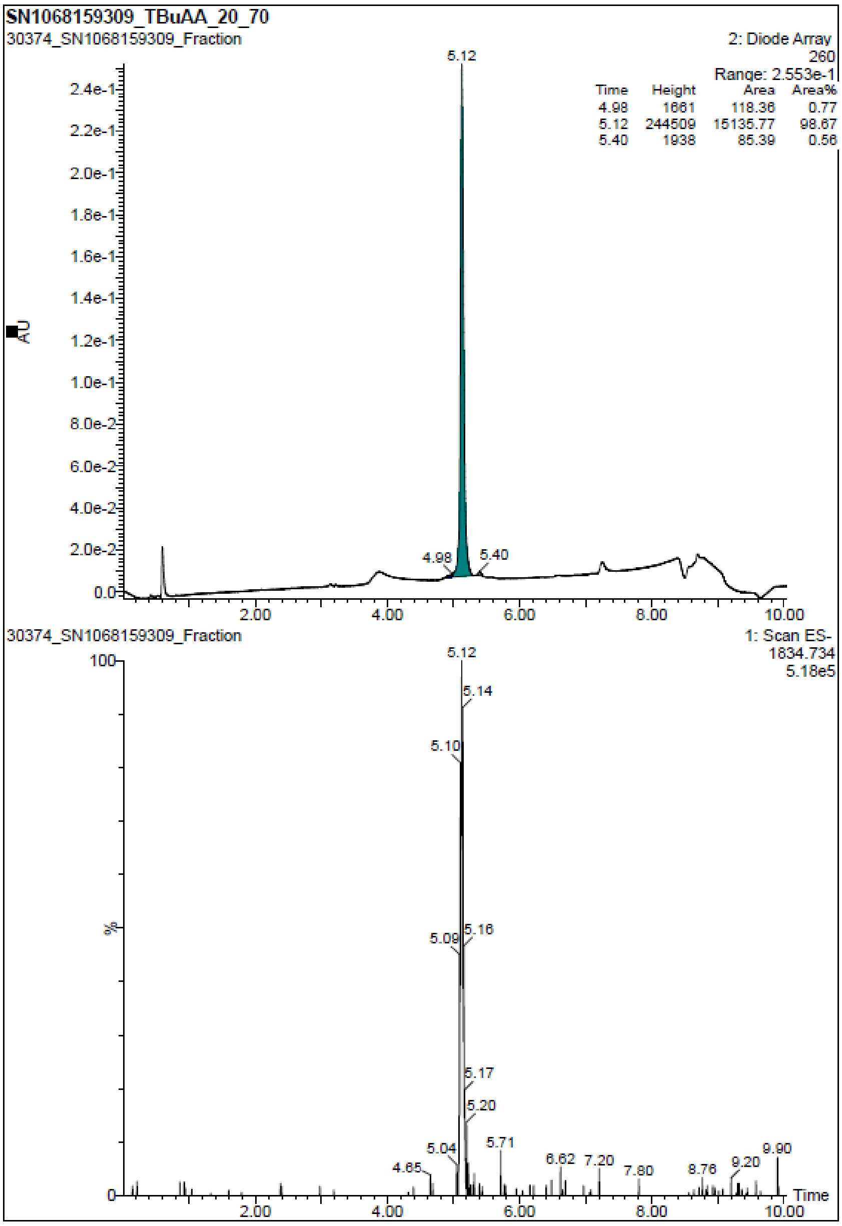

Figure S3. HPLC-MS and HRMS for gapmer tC-1, obtained as a yellow powder using HPLC method A (NH_3 salt, 9 %); UV purity 99%; *m/z* calcd 5502.6153; found: 5502.6305.

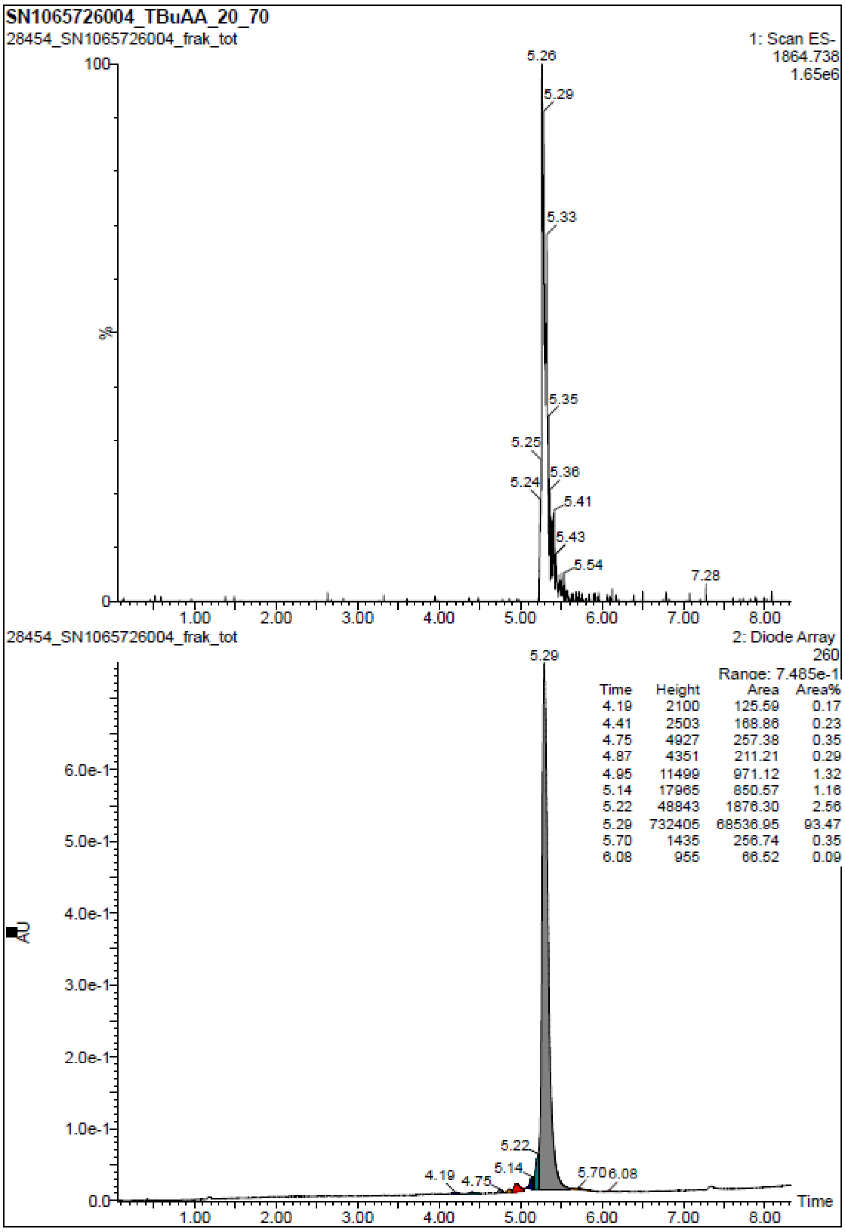

Figure S4. HPLC-MS and HRMS for gapmer tC-2, obtained as a yellow powder using HPLC method A (NH_3 salt, 38%); UV purity 93%; *m*/*z* calcd 5594.5873; found: 5594.5875.

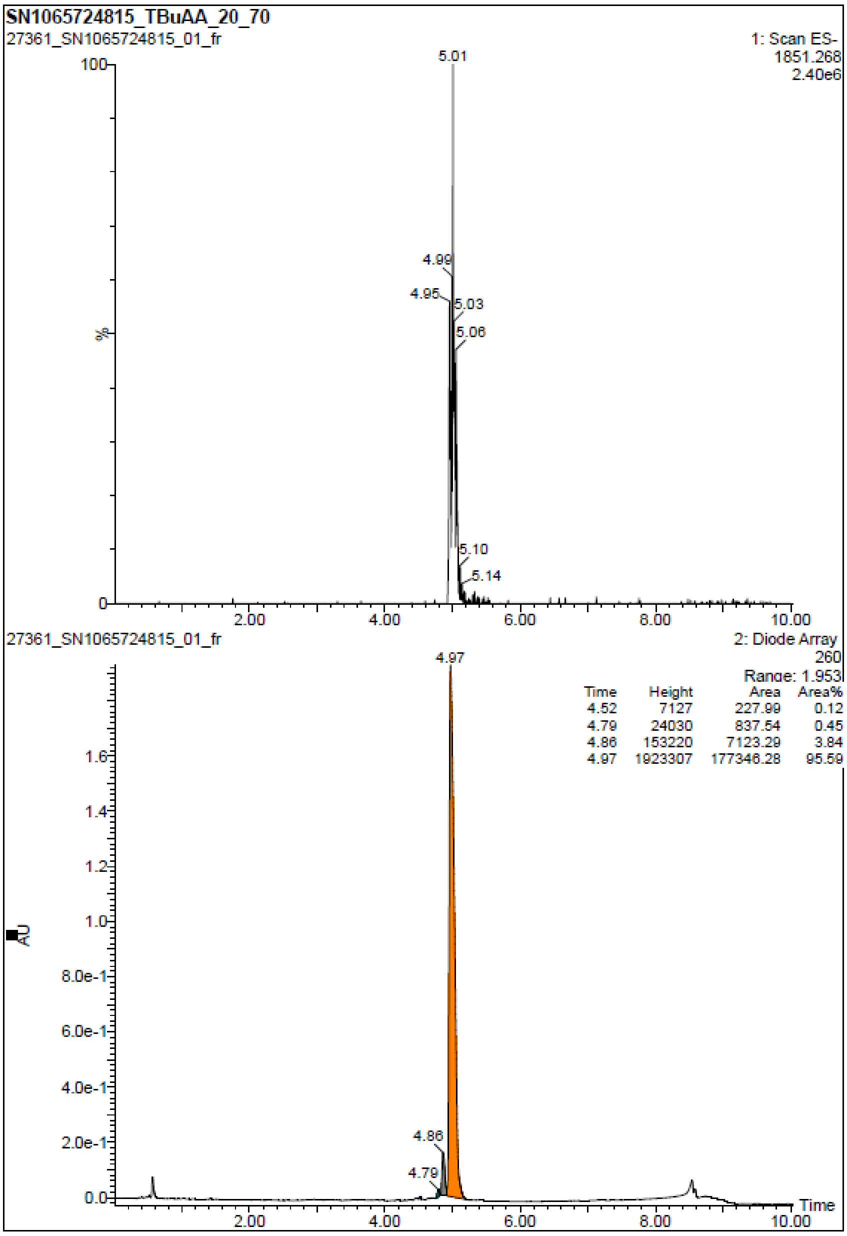

Figure S5. HPLC-MS and HRMS for gapmer tC-2[w], obtained as a yellow powder using HPLC method A (NH_3 salt, 35%); UV purity 96%; *m*/*z* calcd 5552.5768; found: 5552.5761.

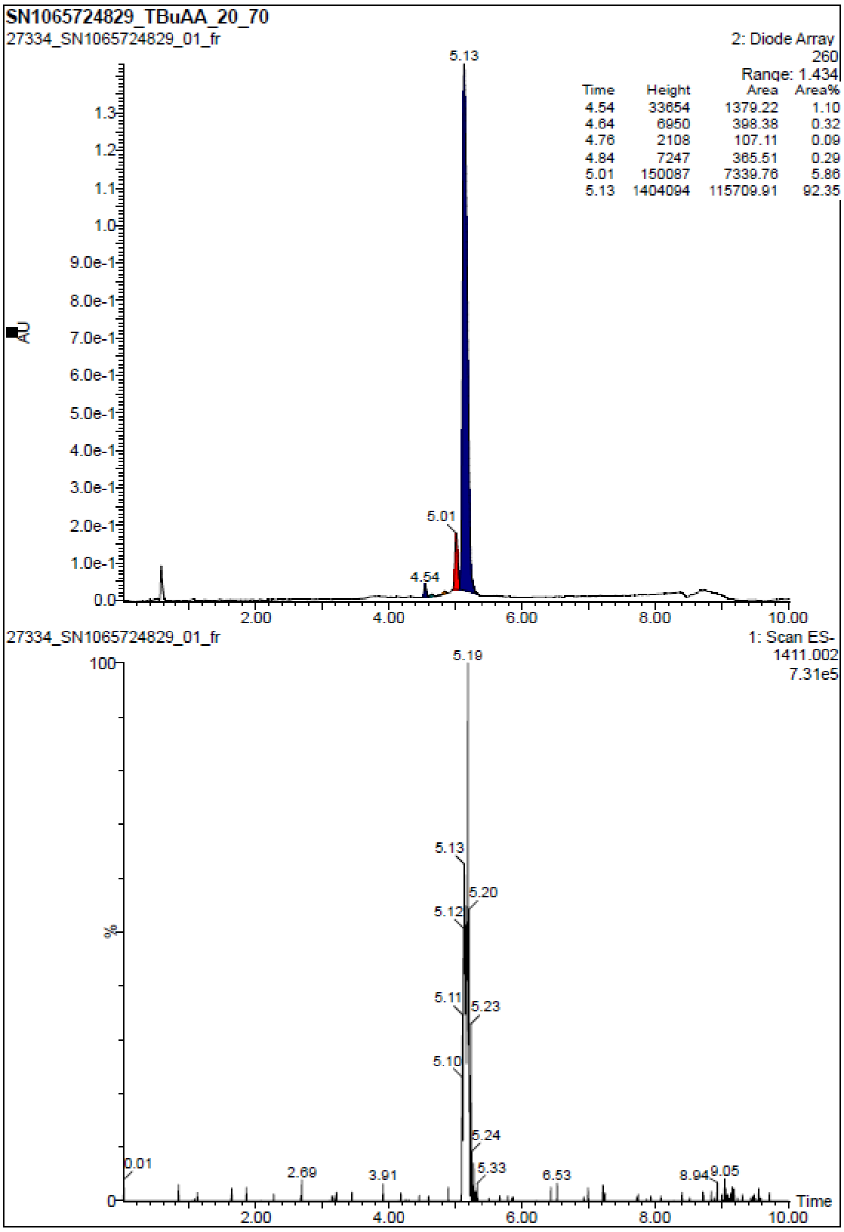

Figure S6. HPLC-MS and HRMS for gapmer tC-3, obtained as a yellow powder using HPLC method A (NH_3 salt, 35%); UV purity 92%; *m/z* calcd 5644.5488; found: 5644.5501.

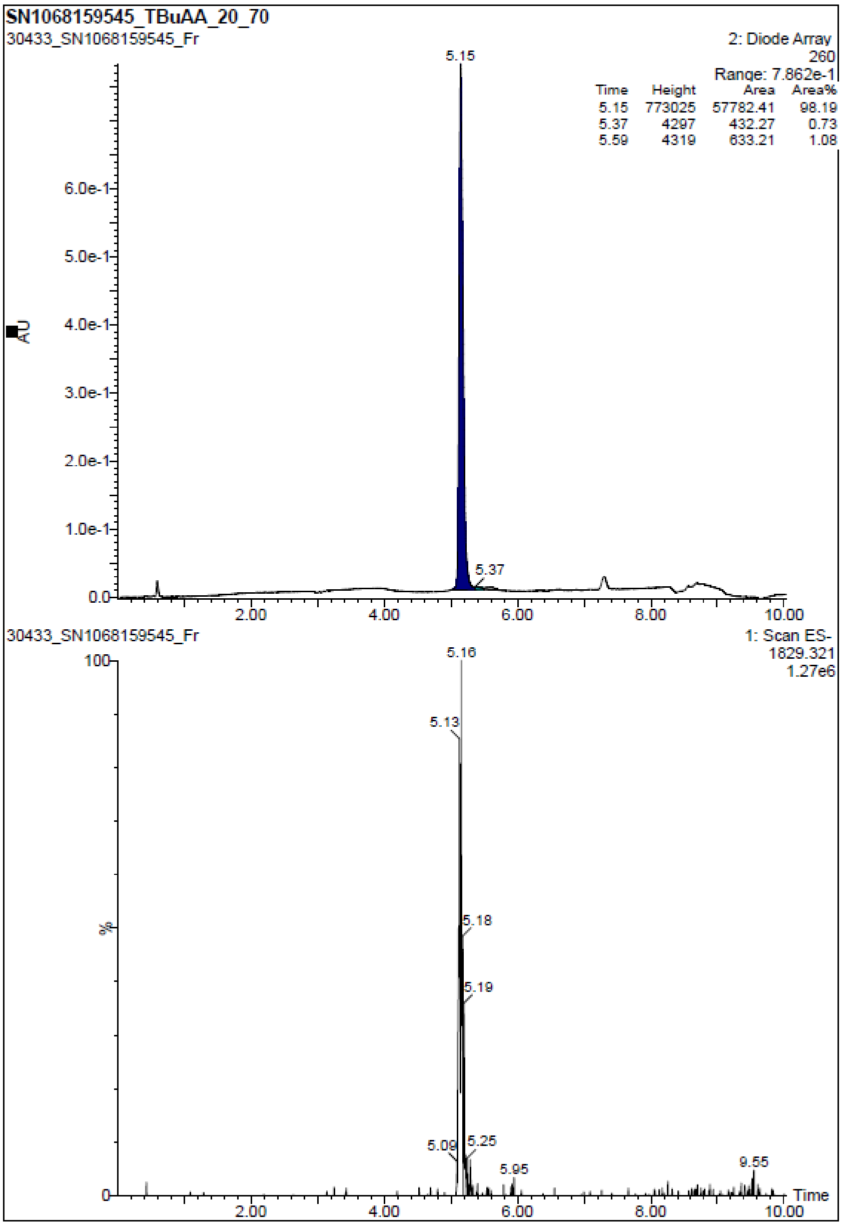

Figure S7. HPLC-MS and HRMS for gapmer tC^{O}-1, obtained as an off-white powder using HPLC method A (NH_3 salt, 30 %); UV purity 98%; *m*/*z* calcd 5486.6381; found: 5486.7843.

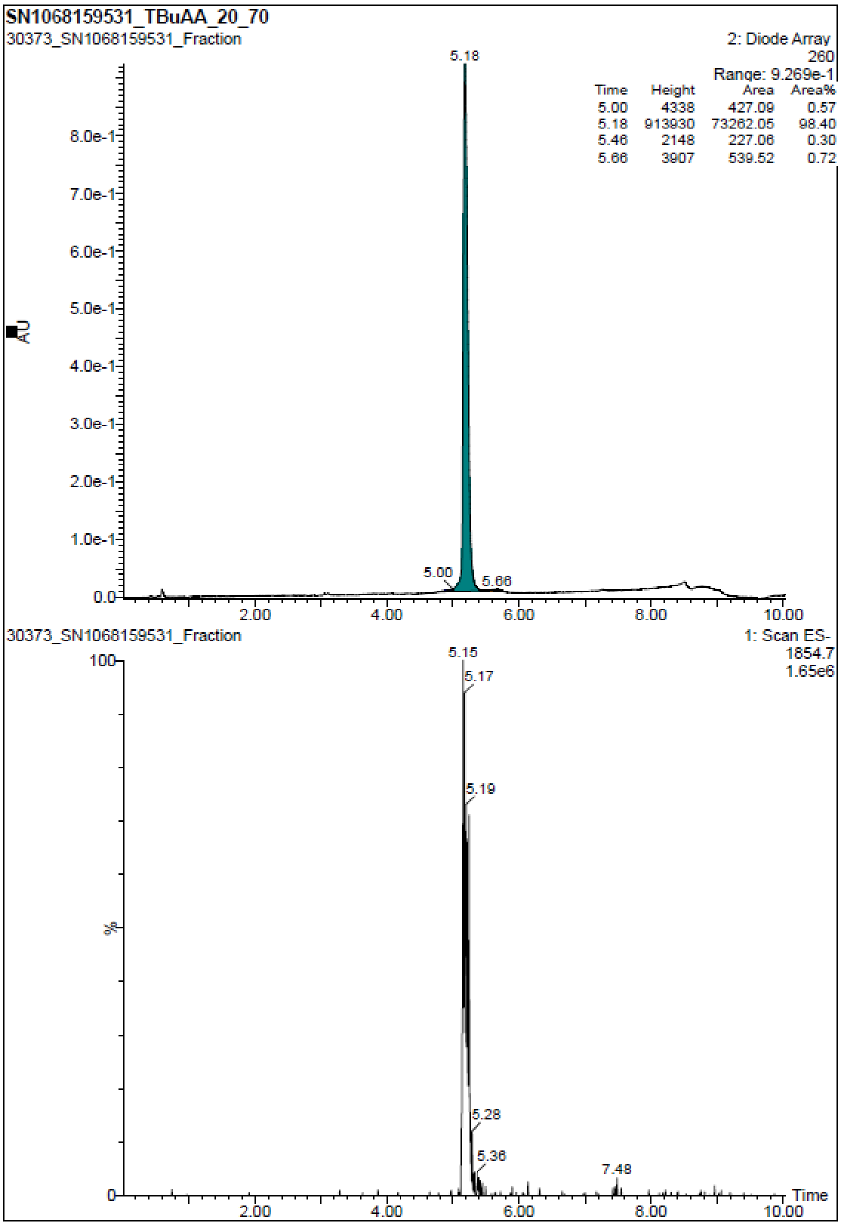

Figure S8. HPLC-MS and HRMS for gapmer tC^{O}-2, obtained as an off-white powder using HPLC method A (NH_3 salt, 34 %); UV purity 98%; *m/z* calcd 5562.6330; found: 5562.7159.

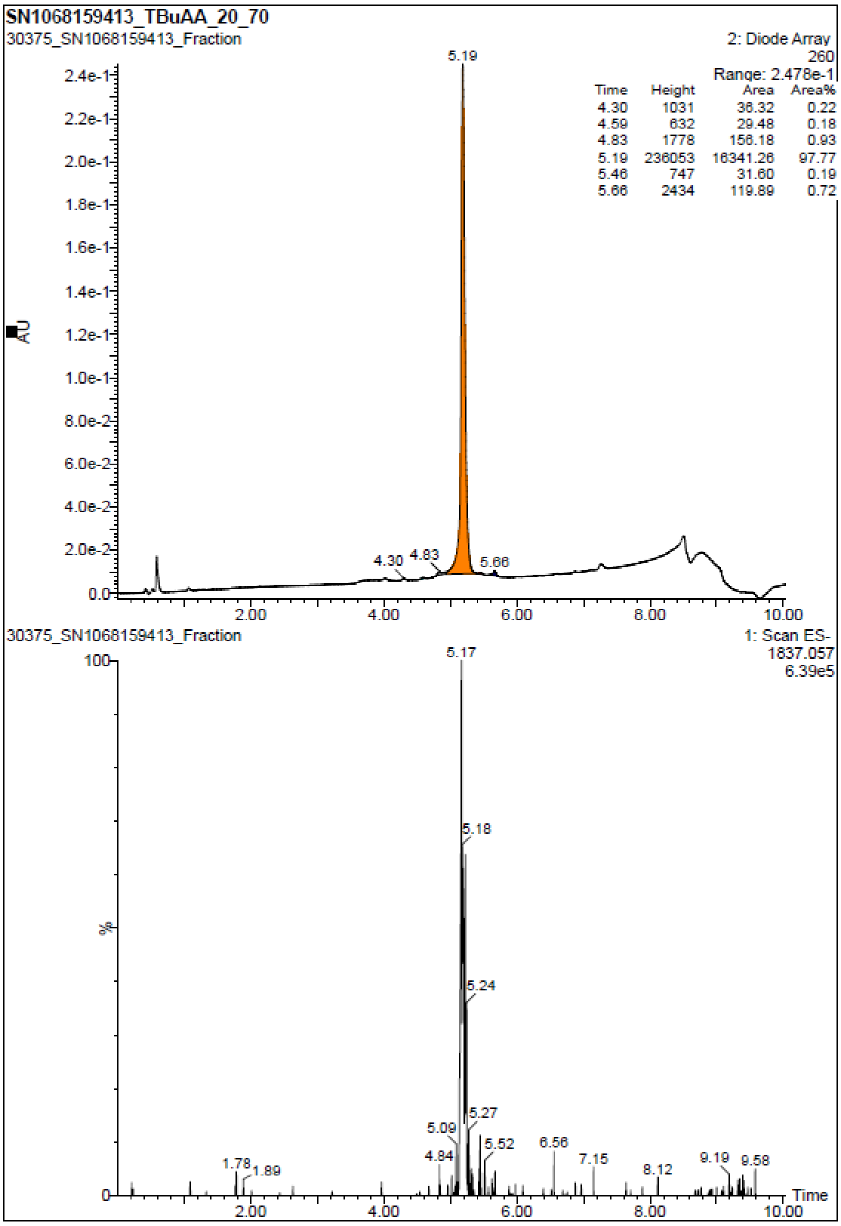

Figure S9. HPLC-MS and HRMS for gapmer 2CNqA-1, obtained as an off-white powder using HPLC method A (NH_3 salt, 9%); UV purity 98%; *m/z* calcd 5508.6589; found: 5508.5787.

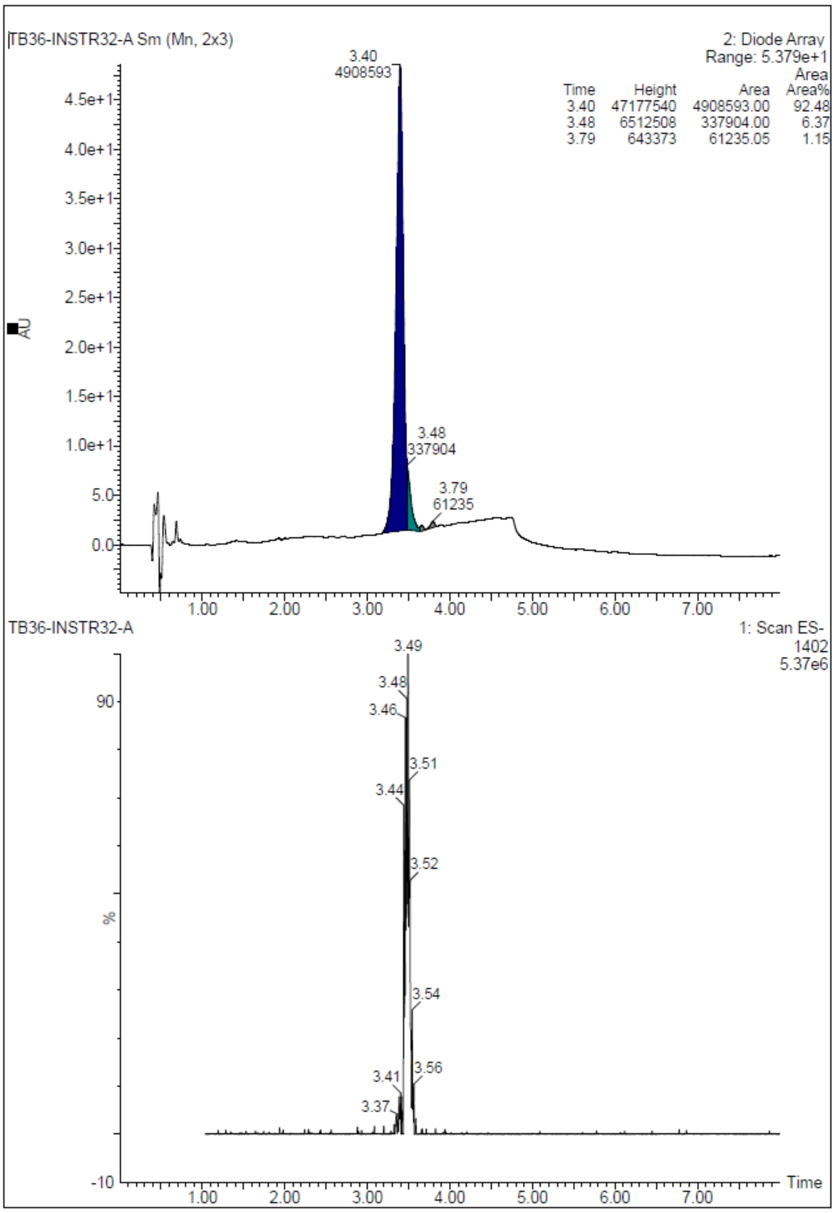

Figure S10. HPLC-MS and HRMS for gapmer 2CNqA-2, obtained as an off-white powder using HPLC method A (NH_3 salt, 11 %); UV purity 92%; *m/z* calcd 5606.6745; found: 5606.7436.

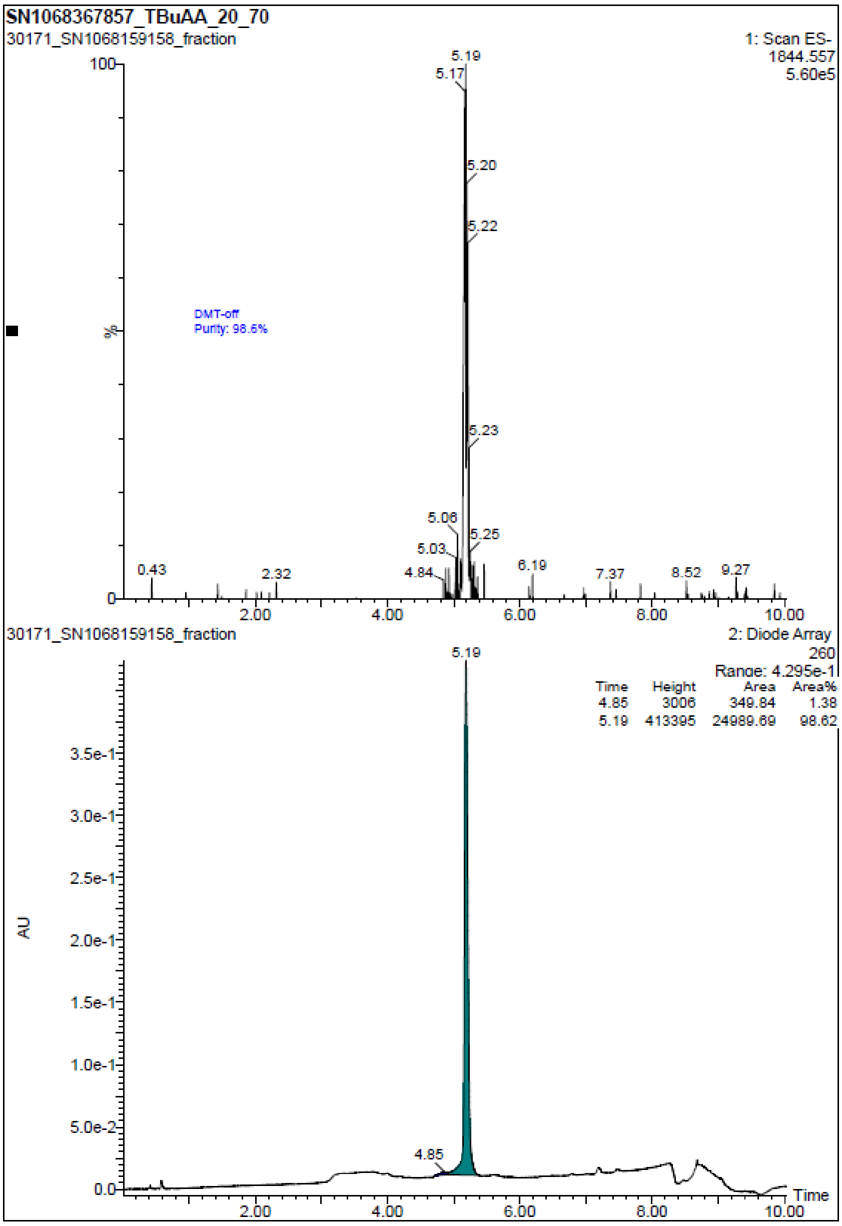

Figure S11. HPLC-MS and HRMS for gapmer pA-1^{TG}, obtained as an off-white powder using HPLC method A (NH_3 salt, 15%); UV purity 99%; *m*/*z* calcd 5533.6793; found: 5533.7288.

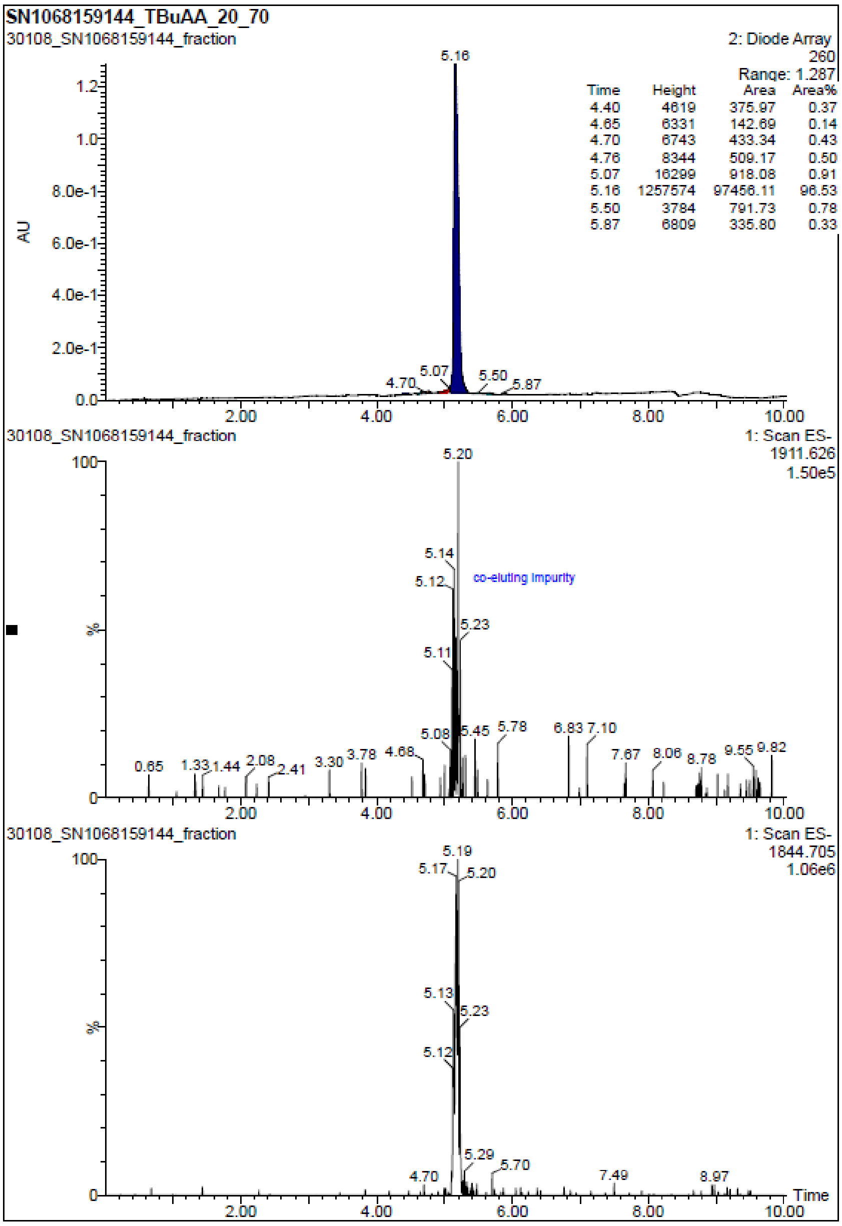

Figure S12. HPLC-MS and HRMS for gapmer pA-1TA, obtained as an off-white powder using HPLC method A (NH_3 salt, 24 %); UV purity 97%; *m*/*z* calcd 5533.6793; found: 5533.8392.

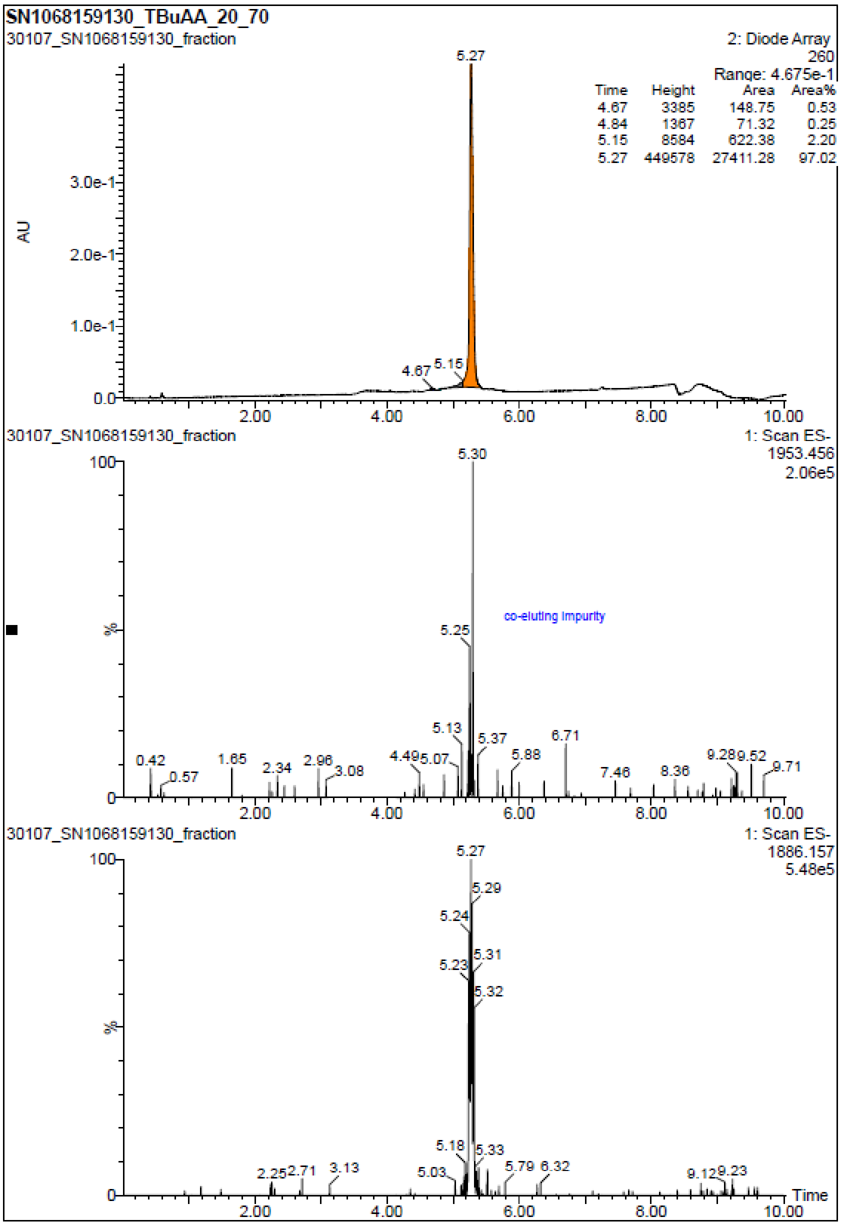

Figure S13. HPLC-MS and HRMS for gapmer pA-2, obtained as an off-white powder using HPLC method A (NH_3 salt, 22 %); UV purity 97%; *m*/*z* calcd 5656.7154; found: 5656.6538.

1.2 Cy3-labeling of gapmers

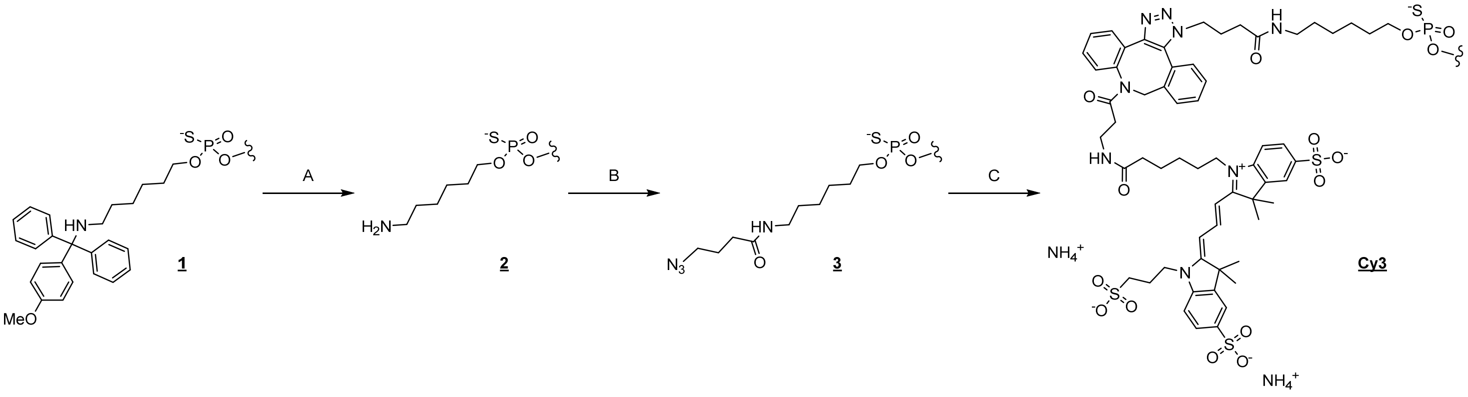

Figure S14. General synthetic scheme for the Cy3-labeled *MALAT1* gapmer.

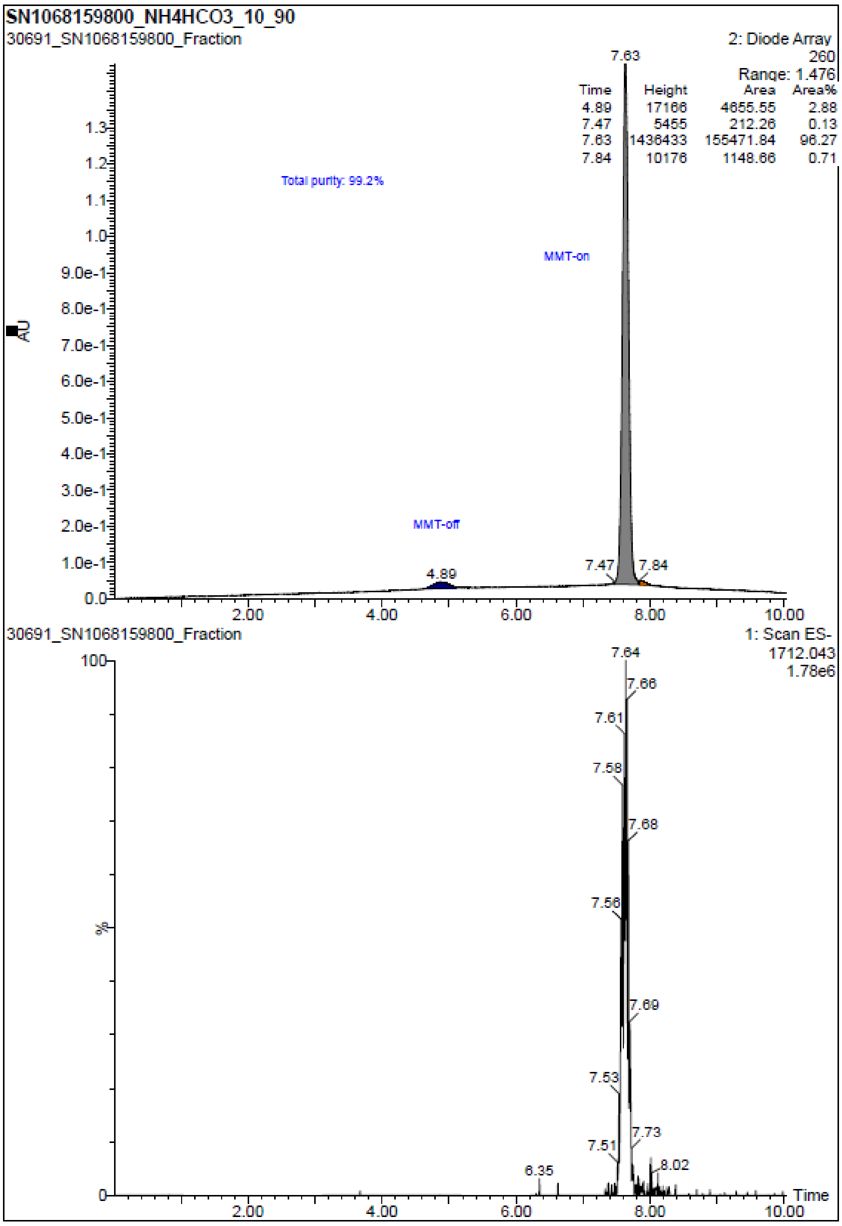

Figure S15. HPLC-MS and HRMS for the 5’-hexylamine-MMT gapmer **1**, preliminary obtained as a white powder using HPLC method B (NH_3 salt, 45%); UV purity 96% (3% deprotected ON was observed at 4.9 min).

A. Removal of the 5'-MMT protecting group:

The 5'-hexylamine-MMT gapmer **1** was solubilized in 2 mL water and 20% acetic acid was added until pH 5. The solution was then warmed to 40 °C for 1 h and 3 M sodium acetate buffer (pH 5.5, 200 µL) was subsequently added. The deprotected gapmer **2** was precipitated in cold ethanol (4 volumes) at -20 °C for 1 h, centrifuged down, and the supernatant was discarded. The pellet was resuspended in cold ethanol, centrifuged down and the supernatant was discarded again. The residue was dried under a flow of argon to allow gapmer **2** (white powder, Na^+ salt, quantitative yield assumed) with no further purification.

B. 5'-N_2 functionalisation of the gapmer:

The 5'-hexylamine-NH2 gapmer **2** (typically 30–50 mg) was taken in a minimal volume of sodium tetraborate buffer (pH 9.1) and a few drops of ACN. Subsequently, 2,5-dioxopyrrolidin-1-yl 4-azidobutanoate (5 equivalents) was added and the reaction was stirred at 20 °C for 15 min. The solution was then neutralized with 3 M sodium acetate buffer (pH 5.5) before cold ethanol was added (4 volumes). Gapmer **3** was precipitated at -20 °C for 1 h, centrifuged down, and the supernatant was discarded. The pellet was resuspended in cold ethanol, centrifuged down and the supernatant was discarded again. The residue was diluted in water with 20% CAN and freeze-dried to allow gapmer **3** (white powder, Na^+ salt, quantitative yield assumed), used as such in the next step without further purification.

C. Cy3-labeling *via* SPAAC:

The 5'-N_2 gapmer **3** (typically 2–5 mg) was dissolved in phosphate buffered saline (pH 7.4, 70 µL) before a solution of Cy3-DBCO (1.1 equivalents) in DMSO (30 µL) was added. The mixture was stirred at 20 °C for 5 min, diluted in water with 20% ACN and freeze-dried. HPLC method C allowed for gapmer Cy3 as a pink powder.

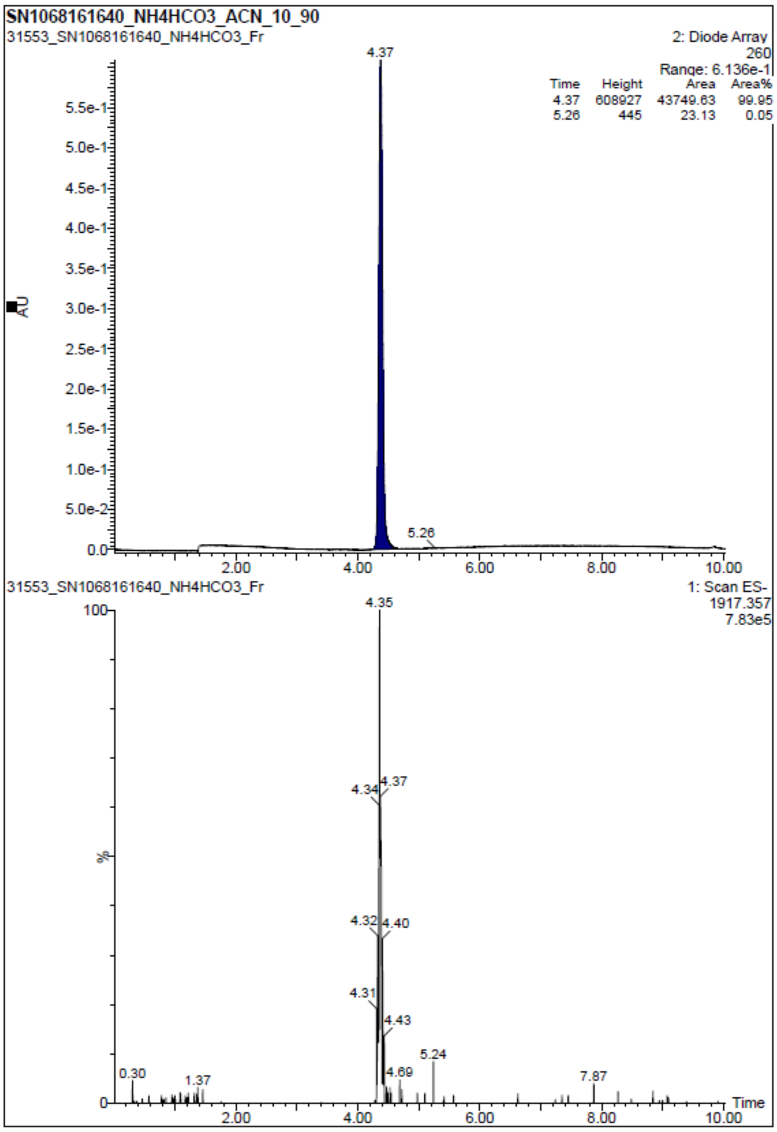

Figure S16. HPLC-MS and HRMS for gapmer Cy3, obtained as a pink powder (NH3 salt, 58%); UV purity 100%; *m/z* calcd 7667.1273; found: 7667.5993.

2 SPECTROSCOPIC CHARACTERIZATION

2.1 Molar absorptivity spectra of FBA-labeled gapmers

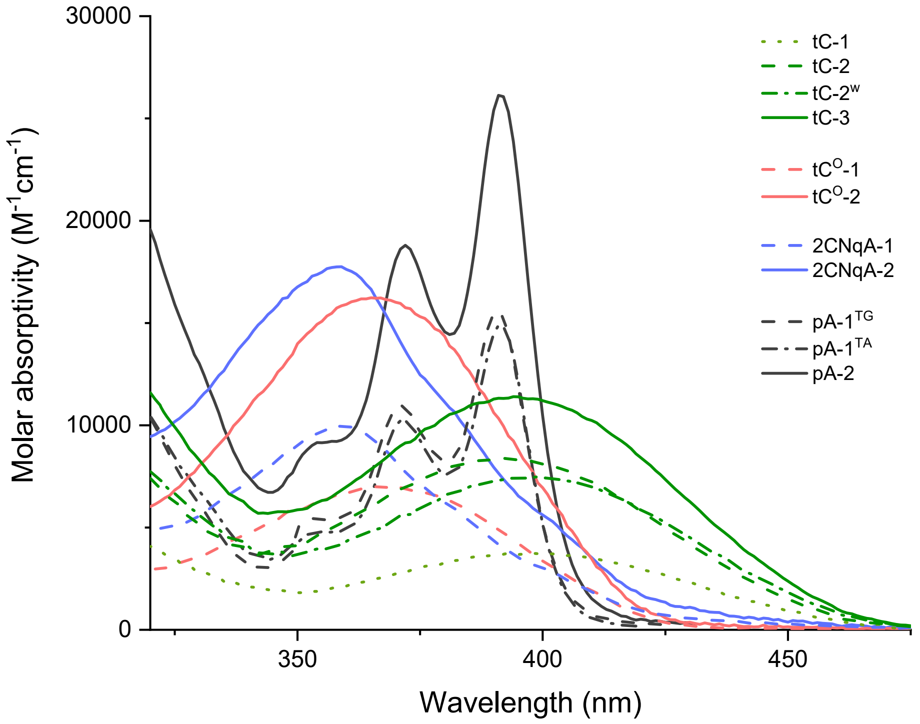

Figure S17. UV-vis molar absorptivity spectra at room temperature (*ca.* 22°C) in 10 mM phosphate buffer (pH 7.4) with 100 mM NaCl and 1.0 mM EDTA added, showing the FBA band of the FBA-modified gapmers. The spectra were afforded by scaling the absorption spectrum of each gapmer to its calculated molar absorptivity at 260 nm (see Methods section in manuscript for calculation of molar absorptivity).

2.2 Brightness of FBA-labeled gapmers

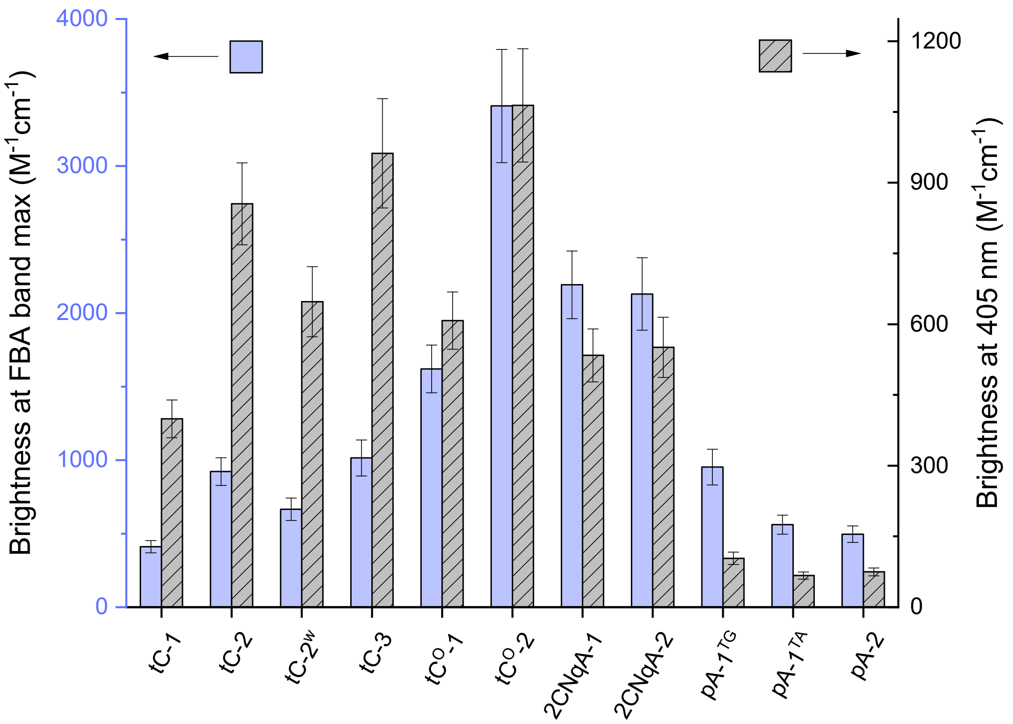

Figure S18. Brightness ($\Phi_F \times \varepsilon(\lambda)$, mean ± standard deviation) of the single-stranded FBA-labeled gapmers at the corresponding FBA band maximum (left scale, blue bars) and at λ = 405 nm (excitation wavelength in the microscopy and cytometry experiments; right scale, gray striped bars), in 10 mM phosphate buffer (pH 7.4) with 100 mM NaCl and 1.0 mM EDTA added. Molar absorptivities, for which a 10% standard deviation is assumed, were taken from Supplementary Fig. S17. A wavelength-independent fluorescence quantum yield is assumed.

2.3 Absorption, emission, and fluorescence decay data in the absence and presence of RNA

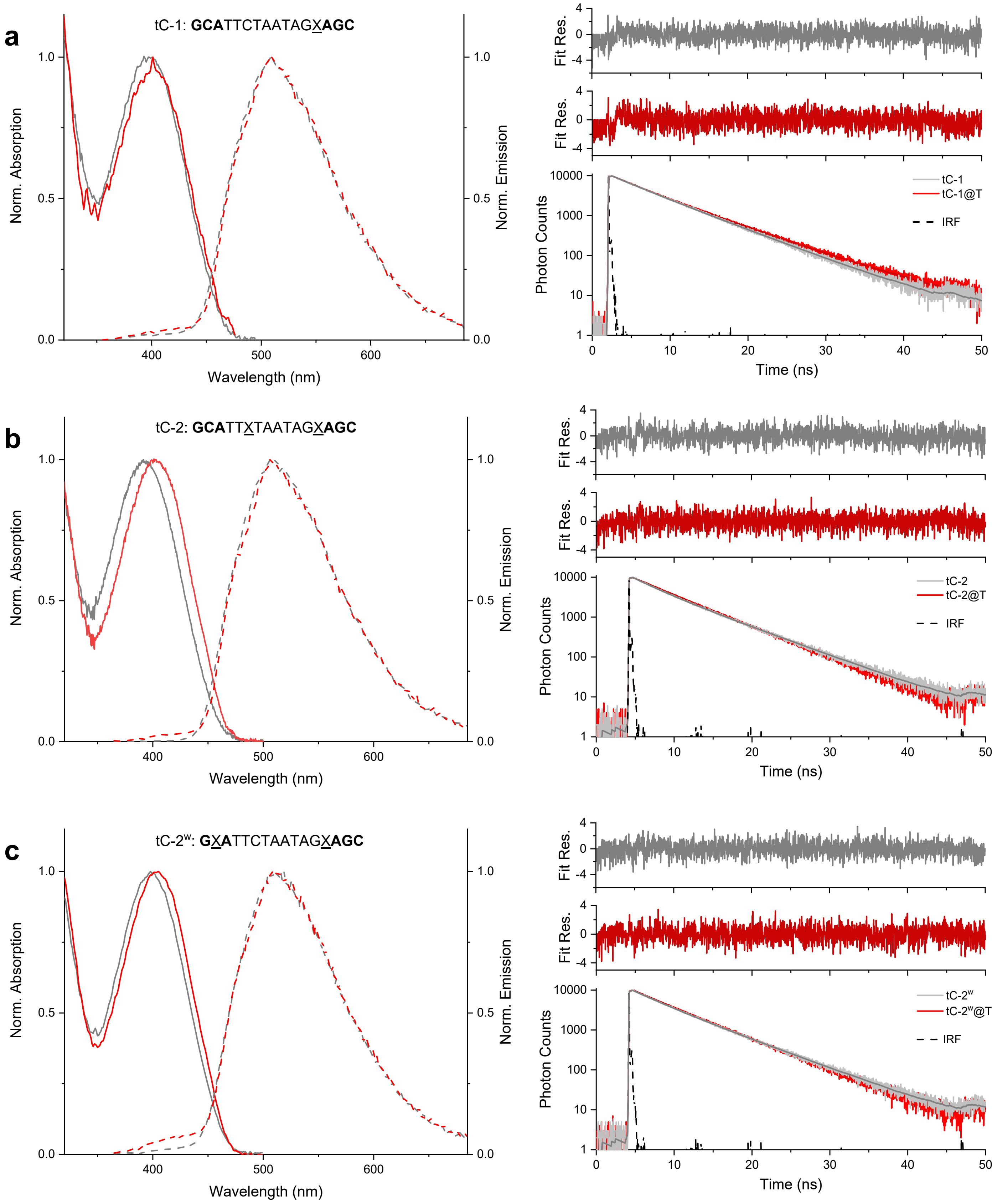

Figure S19. Spectroscopic characterization of the gapmer (gray) and gapmer:RNA duplex (red) in 10 mM phosphate buffer (pH 7.4) with 100 mM NaCl and 1.0 mM EDTA added. Left panel: UV-vis absorption (solid lines) and emission (dashed lines) spectra normalized to 1 at the FBA band maxima. Right bottom panel: Time-correlated single photon counting. Fitted curves for the gapmer (dark gray line) and gapmer:RNA duplex (dark red line), and the instrument response function (IRF, dashed black line) are shown. Right top panels: Residuals for the exponential fits. Fitting parameters are provided in Table S1.

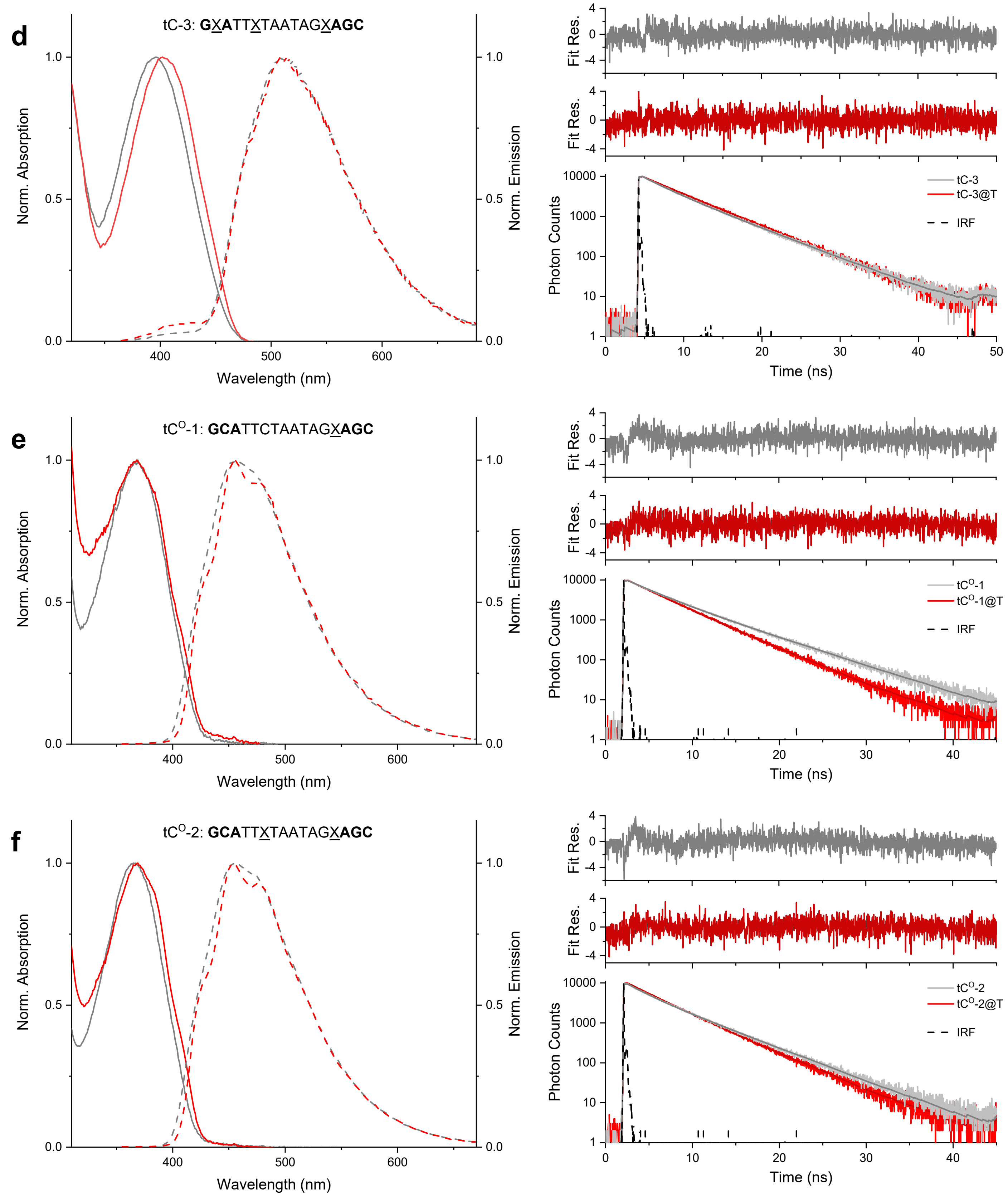

Figure S19 (continued). Spectroscopic characterization of the gapmer (gray) and gapmer:RNA duplex (red) in 10 mM phosphate buffer (pH 7.4) with 100 mM NaCl and 1.0 mM EDTA added. Left panel: UV-vis absorption (solid lines) and emission (dashed lines) spectra normalized to 1 at the FBA band maxima. Right bottom panel: Time-correlated single photon counting. Fitted curves for the gapmer (dark gray line) and gapmer:RNA duplex (dark red line), and the instrument response function (IRF, dashed black line) are shown. Right top panels: Residuals for the exponential fits. Fitting parameters are provided in Table S1.

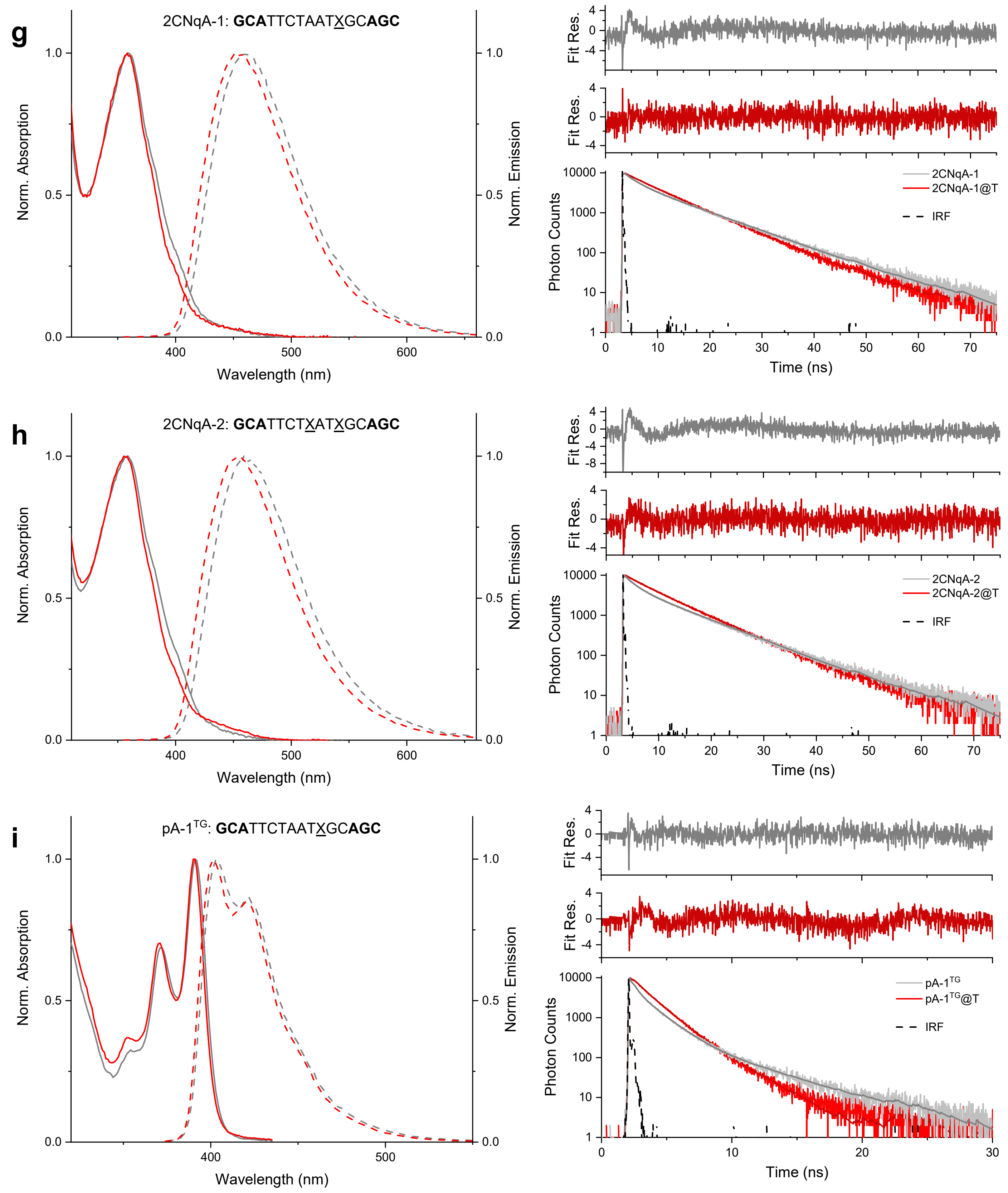

Figure S19 (continued). Spectroscopic characterization of the gapmer (gray) and gapmer:RNA duplex (red) in 10 mM phosphate buffer (pH 7.4) with 100 mM NaCl and 1.0 mM EDTA added. Left panel: UV-vis absorption (solid lines) and emission (dashed lines) spectra normalized to 1 at the FBA band maxima. Right bottom panel: Time-correlated single photon counting. Fitted curves for the gapmer (dark gray line) and gapmer:RNA duplex (dark red line), and the instrument response function (IRF, dashed black line) are shown. Right top panels: Residuals for the exponential fits. Fitting parameters are provided in Table S1.

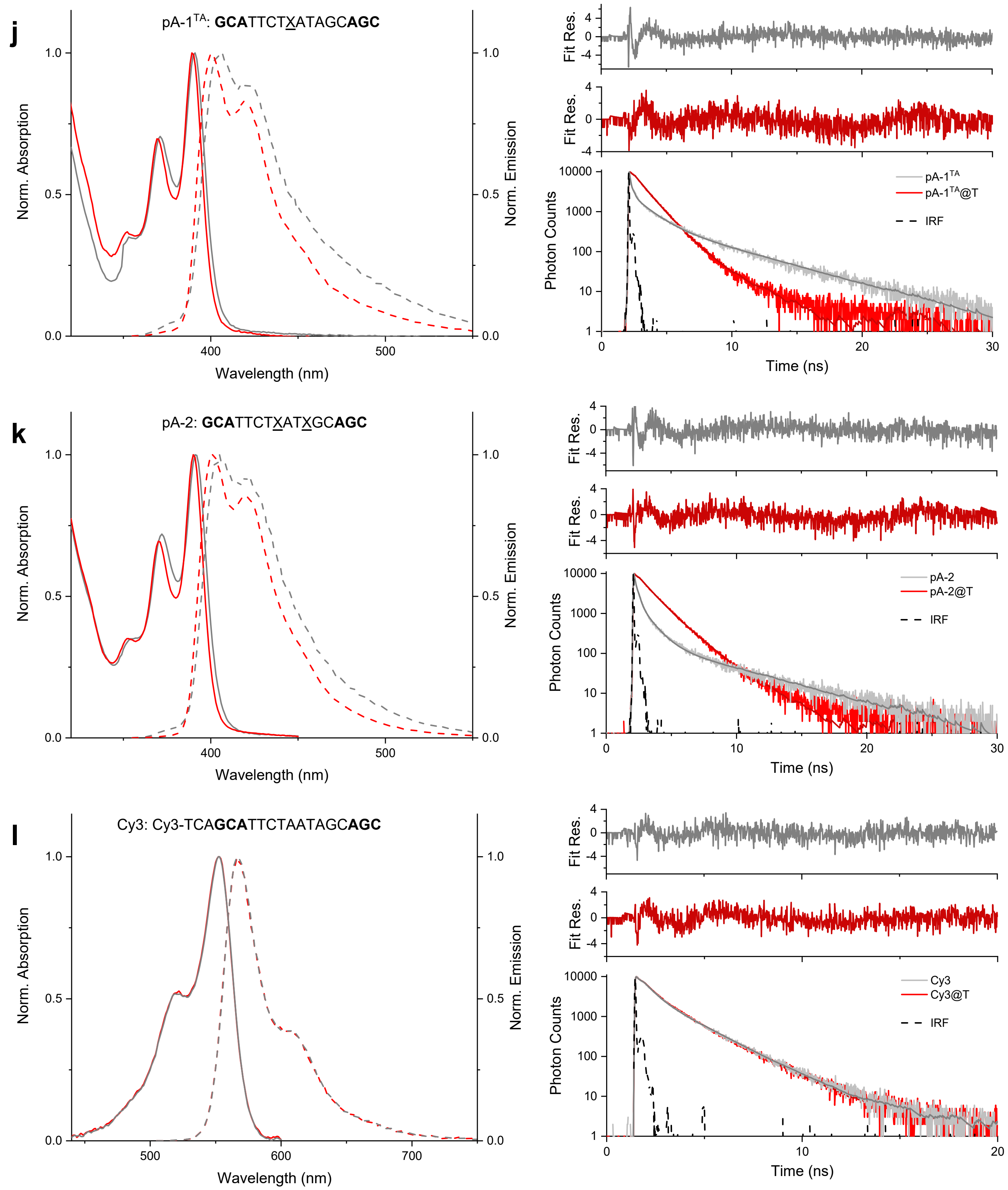

Figure S19 (continued). Spectroscopic characterization of the gapmer (gray) and gapmer:RNA duplex (red) in 10 mM phosphate buffer at pH 7.4 with 100 mM NaCl and 1.0 mM EDTA added. Left panel: UV-vis absorption (solid lines) and emission (dashed lines) spectra normalized to 1 at the FBA band maxima. Right bottom panel: Time-correlated single photon counting. Fitted curves for the gapmer (dark gray line) and gapmer:RNA duplex (dark red line), and the instrument response function (IRF, dashed black line) are shown. Right top panels: Residuals for the exponential fits. Fitting parameters are provided in Table S1.

2.4 Fitting of fluorescence lifetimes

The time-resolved single photon counting intensity decays were fitted with IRF re-convolution to the multiexponential model:

$$I(t) = \int_0^t IRF(t') \sum_{i=1}^{n} \alpha_i e^{-\frac{t-t'}{\tau_i}} dt'$$

The least-square re-convolution fitting procedure was carried out by the DecayFit software (DecayFit - Fluorescence Decay Analysis Software 1.3, FluorTools, www.fluortools.com). The decays were fitted to either a bi- or tri-exponential (n = 2 or 3) model. The presented lifetimes are amplitude-weighted lifetimes ($\bar{\tau}$), calculated according to:

$$\bar{\tau} = \sum_{i=1}^{n} \alpha_i \tau_i$$

The fitted parameters are collected in Table S1.

Table S1. Fluorescence lifetime and fluorescence quantum yield of the labeled gapmers. The fluorescence lifetime fitting parameters α_i and τ_i presented here were calculated from a representative experimental replicate. The "@T" suffix denotes gapmers hybridized to the fully complementary RNA target sequence T.

Gapmer	α_1	τ_1	α_2	τ_2	α_3	τ_3	$\bar{\tau}^a$	X^{2b}	$\tau_F^{c,d}$	$\Phi_F^{d,e}$
tC-1	0.519	4.30	0.481	7.11	-	-	5.7	1.08	5.6±0.03	11±0.1
tC-1@T	0.376	4.24	0.624	6.24	-	-	5.5	1.13	5.5±0.02	8.0±0.5
tC-2	0.335	2.94	0.665	6.10	-	-	5.0	1.07	5.0±0.02	11±0.2
tC-2@T	0.0649	2.31	0.935	5.48	-	-	5.3	1.00	5.3±0.001	8.5±0.2
tC-2^w	0.327	3.44	0.673	6.09	-	-	5.2	1.07	5.2±0.002	8.9±0.5
tC-2^w@T	0.0641	2.29	0.936	5.50	-	-	5.3	1.05	5.3±0.01	7.1±0.01
tC-3	0.321	2.77	0.679	5.80	-	-	4.8	1.09	4.8±0.006	8.9±0.6
tC-3@T	0.0834	2.72	0.917	5.49	-	-	5.3	1.07	5.3±0.01	7.9±0.1
tC^O-1	0.429	3.19	0.571	6.19	-	-	4.9	1.20	4.9±0.02	23±0.1
tC^O-1@T	0.835	4.02	0.166	5.74	-	-	4.3	1.06	4.3±0.05	15±0.2
tC^O-2	0.332	2.21	0.668	5.19	-	-	4.2	1.32	4.2±0.02	21±1.1
tC^O-2@T	0.454	3.37	0.546	4.78	-	-	4.1	1.08	4.1±0.04	17±0.6
2CNqA-1	0.423	2.35	0.577	9.35	-	-	6.4	1.38	6.2±0.2	22±0.7
2CNqA-1@T	0.214	4.25	0.789	7.96	-	-	7.2	1.12	7.1±0.05	21±0.2
2CNqA-2	0.501	2.05	0.499	8.66	-	-	5.3	1.64	5.3±0.1	12±0.7
2CNqA-2@T	0.234	3.32	0.766	7.56	-	-	6.6	1.22	6.5±0.07	15±0.04
pA-1^{TG}	0.521	0.345	0.445	1.51	0.0342	4.73	1.0	1.05	1.0±0.01	6.4±0.5
pA-1^{TG}@T	0.669	1.14	0.331	2.10	-	-	1.5	1.23	1.5±0.02	8.6±0.6
pA-1TA	0.807	0.126	0.152	1.29	0.0406	4.83	0.49	1.24	0.52±0.03	3.6±0.2
pA-1TA@T	0.892	1.01	0.108	2.00	-	-	1.1	1.06	1.1±0.01	7.4±0.4
pA-2	0.825	0.172	0.161	0.882	0.0146	4.88	0.36	1.08	$0.35\pm6\times10^{-5}$	1.9±0.1
pA-2@T	0.677	0.959	0.323	1.76	-	-	1.2	1.12	1.2±0.01	7.1±0.1
Cy3	0.679	0.545	0.321	1.76	-	-	0.94	1.18	0.97±0.03	19±1.0
Cy3@T	0.647	0.550	0.353	1.69	-	-	0.95	1.19	0.97±0.02	19±0.7

[a] Amplitude-weighted fluorescence lifetime based on the fitting parameters presented in the table. [b] Chi squared (X^2) goodness of fit, see Supplementary Fig. S19 for decay data and fitted curves. [c] Average fluorescence lifetime. [d] Presented values are mean ± standard deviation of two independent experiments.[e] Average fluorescence quantum yield.

2.5 Circular dichroism of gapmer:RNA duplexes

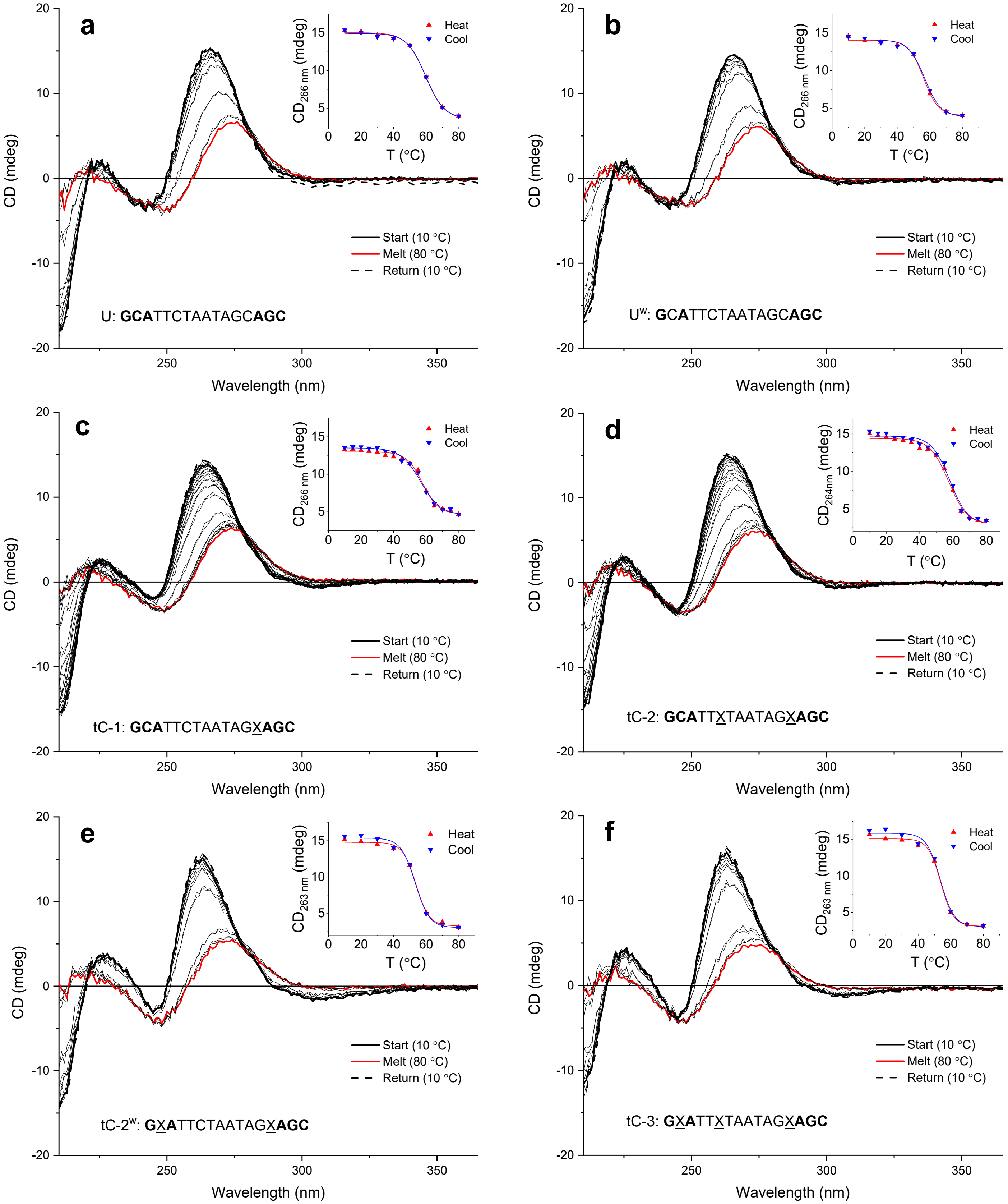

Figure S20. Circular dichroism (CD) spectra of gapmer:RNA duplexes (4 µM) in 10 mM phosphate buffer (pH 7.4) with 100 mM NaCl and 1.0 mM EDTA added, collected during one melting cycle. Inset: CD at λ_{max} (263–267 nm) *vs.* temperature (T) during the heating (red triangles) and cooling (blue triangles) stages. Boltzmann sigmoid fits of the heating (red line) and cooling (blue line) stages are included.

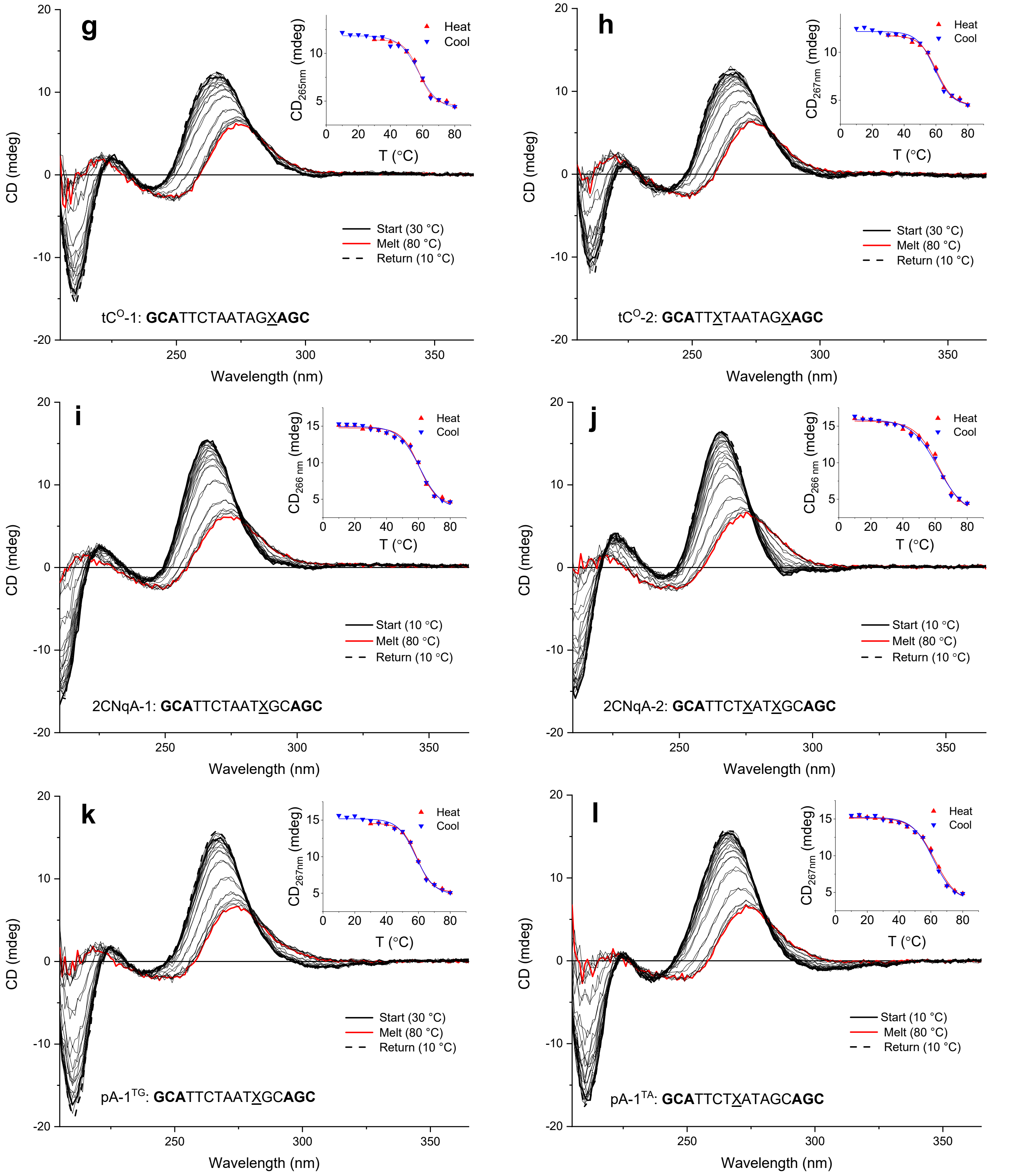

Figure S20 (continued). Circular dichroism (CD) spectra of gapmer:RNA duplexes (4 μM) in 10 mM phosphate buffer (pH 7.4) with 100 mM NaCl and 1.0 mM EDTA added, collected during one melting cycle. Inset: CD at λ_{max} (263–267 nm) *vs.* temperature (T) during the heating (red triangles) and cooling (blue triangles) stages. Boltzmann sigmoid fits of the heating (red line) and cooling (blue line) stages are included.

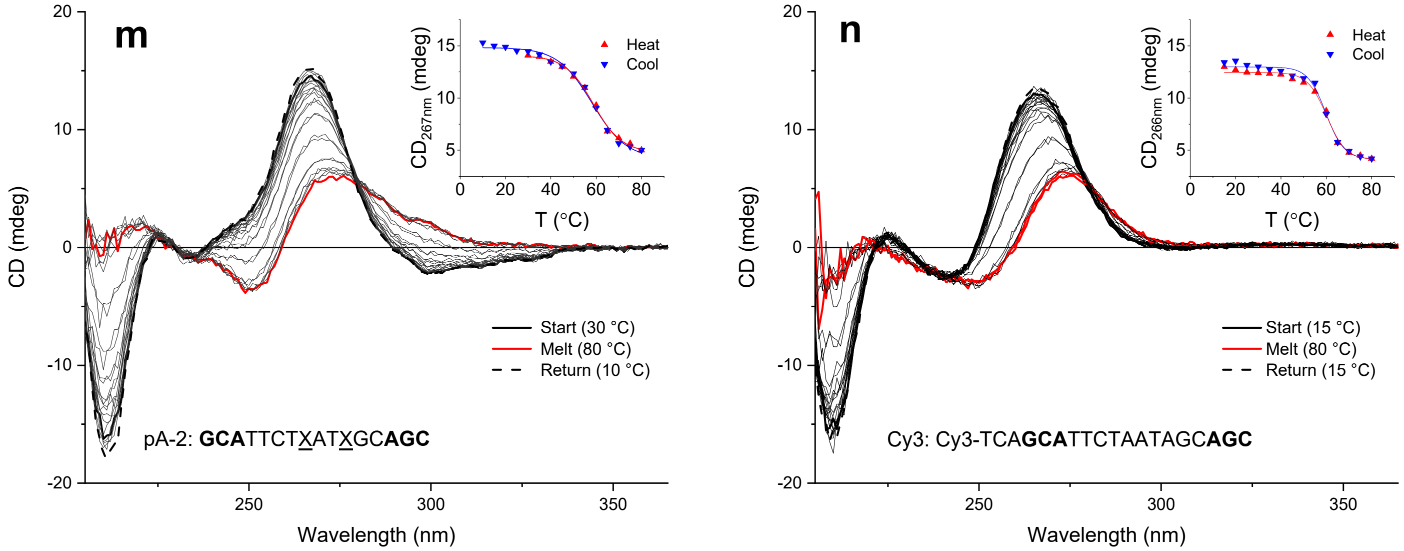

Figure S20 (continued). Circular dichroism (CD) spectra of gapmer:RNA duplexes (4 μM) in 10 mM phosphate buffer (pH 7.4) with 100 mM NaCl and 1.0 mM EDTA added, collected during one melting cycle. Inset: CD at λ_{max} (263–267 nm) *vs.* temperature (T) during the heating (red triangles) and cooling (blue triangles) stages. Boltzmann sigmoid fits of the heating (red line) and cooling (blue line) stages are included.

Table S2. Gapmer:RNA melting temperature (CD T_m) and difference in CD T_m (CD ΔT_m) referenced to the unmodified gapmer U, in 10 mM phosphate buffer (pH 7.4) with 100 mM NaCl and 1.0 mM EDTA added, determined using circular dichroism. Presented values are mean ± standard deviation of the two melting transitions shown in Supplementary Fig. S20.

Gapmer	CD T_m (°C)[a]	CD ΔT_m (°C)
U	59.7 ± 0.01	0 ± 0.01
U^w	56.5 ± 0.4	-3.2 ± 0.5
tC-1	57.2 ± 0.6	-2.5 ± 0.8
tC-2	57.7 ± 0.3	-2.0 ± 0.5
tC-2^w	53.5 ± 0.2	-6.2 ± 0.3
tC-3	53.8 ± 0.1	-5.9 ± 0.1
tC^O-1	57.7 ± 0.2	-2.0 ± 0.2
tC^O-2	59.7 ± 0.6	0.0 ± 0.8
2CNqA-1	60.6 ± 0.2	0.9 ± 0.3
2CNqA-2	62.1 ± 0.3	2.4 ± 0.5
pA-1^{TG}	58.8 ± 0.3	-0.9 ± 0.4
pA-1TA	62.0 ± 0.6	2.3 ± 0.8
pA-2	58.5 ± 0.3	-1.2 ± 0.5
Cy3	60.0 ± 0.2	0.3 ± 0.3

[a] Note that the CD experiments produce T_m that overall are slightly shifted (*ca* -2 °C) compared to those attained in the corresponding absorption experiments (see Table 1 and Table S3). We believe this is due to differences in temperature probing (indirect thermo-block probing in CD *vs* direct in-sample probing in absorption), possibly in combination with differences in equilibrium hysteresis as a result of the longer hold time at each temperature applied in the CD measurements (see Material and Methods in MS).

2.6 UV-monitored melting of gapmer:RNA duplexes

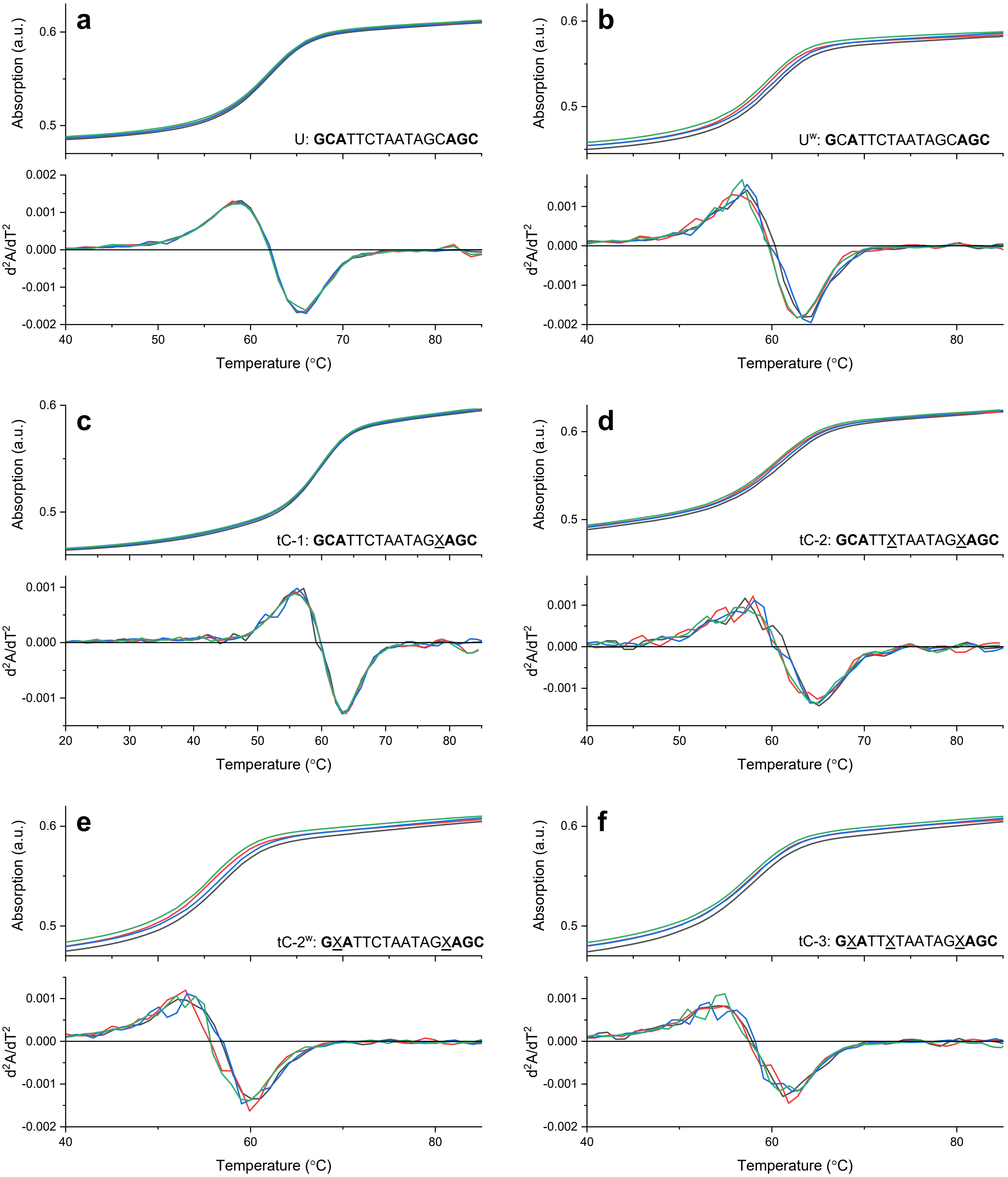

Figure S21. UV monitored melting of gapmer:RNA duplexes (4 μM) in 10 mM phosphate buffer (pH 7.4) with 100 mM NaCl and 1.0 mM EDTA added, monitored during the successive temperature program: 20 °C to 85 °C (black line), 85°C to 20 °C (red line), 20 °C to 85 °C (blue line), and 85 °C to 20 °C (green line). Top panel: Absorption at 260 nm *vs.* temperature. Bottom panel: Second derivative of the absorption (A) as a function of temperature (T*)*. The melting temperatures are collected in Table S3.

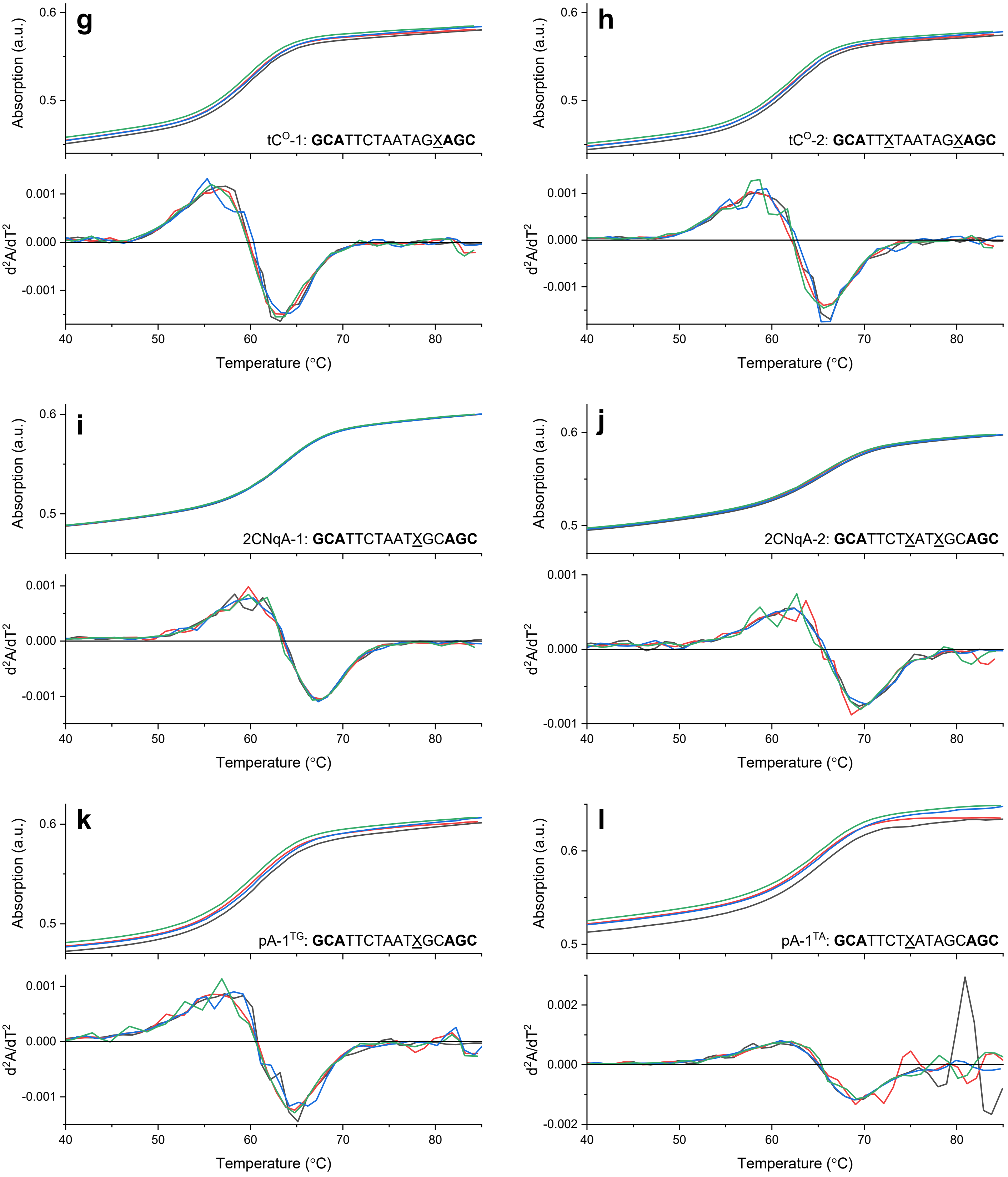

Figure S21 (continued). UV monitored melting of gapmer:RNA duplexes (4 μM) in 10 mM phosphate buffer (pH 7.4) with 100 mM NaCl and 1.0 mM EDTA added, monitored during the successive temperature program: 20 °C to 85 °C (black line), 85°C to 20 °C (red line), 20 °C to 85 °C (blue line), and 85 °C to 20 °C (green line). Top panel: Absorption at 260 nm *vs.* temperature. Bottom panel: Second derivative of the absorption (A) as a function of temperature (T). The melting temperatures are collected in Table S3.

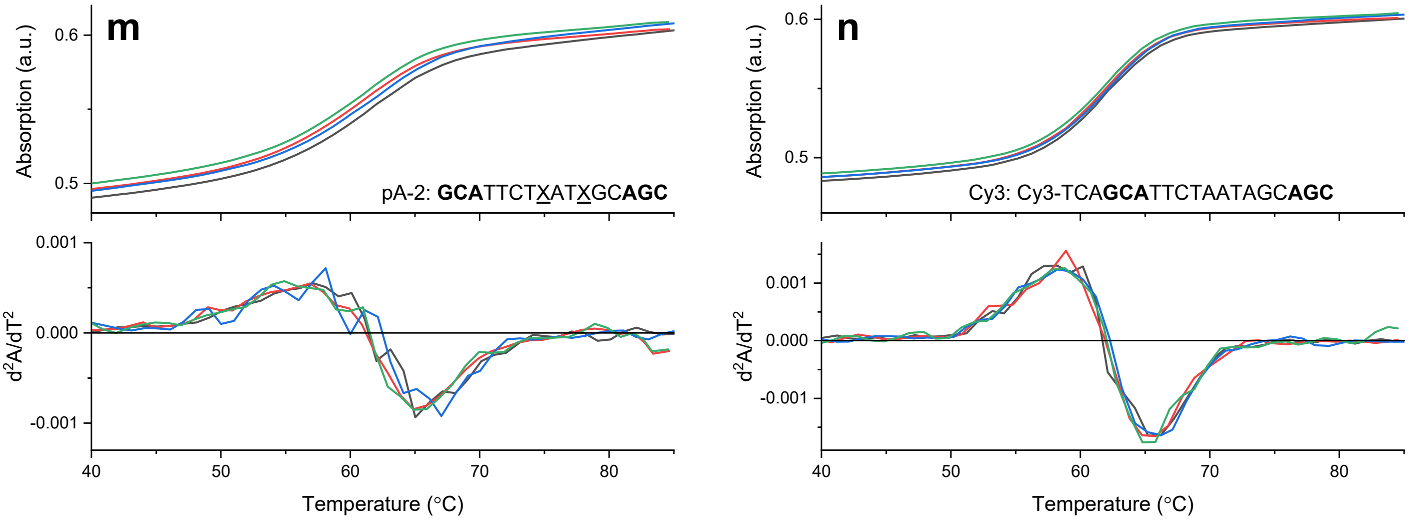

Figure S21 (continued). UV monitored melting of gapmer:RNA duplexes (4 µM) in 10 mM phosphate buffer (pH 7.4) with 100 mM NaCl and 1.0 mM EDTA added, monitored during the successive temperature program: 20 °C to 85 °C (black line), 85°C to 20 °C (red line), 20 °C to 85 °C (blue line), and 85 °C to 20 °C (green line). Top panel: Absorption at 260 nm *vs.* temperature. Bottom panel: Second derivative of the absorption (A) as a function of temperature (T*)*. Melting temperatures are collected in Table S3.

Table S3. Gapmer:RNA melting temperature (T_m) and difference in T_m (ΔT_m) referenced to the unmodified gapmer U, in 10 mM phosphate buffer (pH 7.4) with 100 mM NaCl and 1.0 mM EDTA added, determined using UV-vis absorption. The presented data are mean ± standard deviation of the four melting transitions shown in Supplementary Fig. S21.

Gapmer	T_m (°C)	ΔT_m (°C)
U	61.5 ± 0.4	0 ± 0.5
U^w	59.9 ± 0.3	-1.7 ± 0.5
tC-1	59.7 ± 0.4	-1.8 ± 0.5
tC-2	60.7 ± 0.6	-0.8 ± 0.7
tC-2^w	56.3 ± 0.7	-5.3 ± 0.8
tC-3	57.8 ± 0.3	-3.7 ± 0.5
tC^O-1	60.0 ± 0.3	-1.6 ± 0.4
tC^O-2	62.4 ± 0.3	0.9 ± 0.4
2CNqA-1	63.6 ± 0.2	2.0 ± 0.4
2CNqA-2	65.6 ± 0.2	4.0 ± 0.4
pA-1^{TG}	60.7 ± 0.1	-0.9 ± 0.4
pA-1TA	65.2 ± 0.2	3.6 ± 0.4
pA-2	61.4 ± 0.1	-0.1 ± 0.4
Cy3	62.0 ± 0.2	0.5 ± 0.4

3. BIOLOGICAL EVALUATION

3.1 *MALAT1* knockdown efficiency of the FBA-labeled gapmers

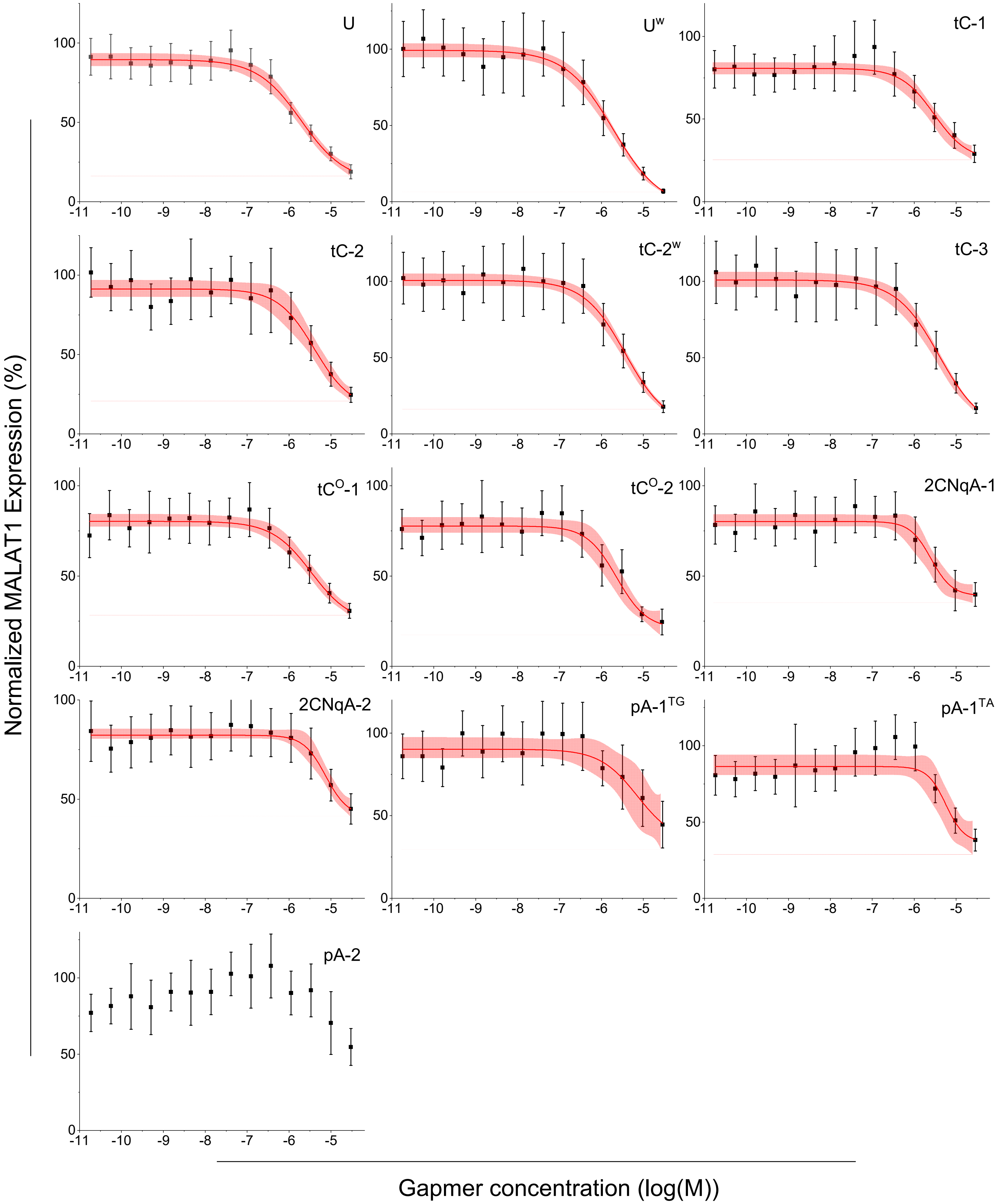

Figure S22. Gapmer-induced *MALAT1* knockdown evaluated using a lysis-based qPCR assay. The *MALAT1* expression levels (black squares, mean ± standard deviation) following exposure to the gapmers were normalized to a negative control (untreated cells). A sigmoidal fit (red line), including a 95% confidence interval (red shaded region), was carried out according to SI Section 3.3. The experiments were conducted in at least two independent replicates with biological duplicates at each occasion.

3.2 *MALAT1* knockdown efficiency of the Cy3-labeled gapmer

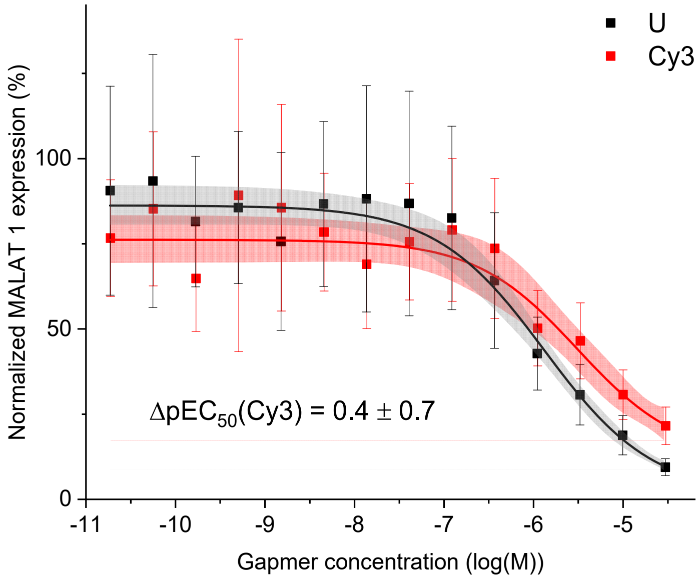

Figure S23. Gapmer-induced *MALAT1* knockdown evaluated using a lysis-based qPCR assay. The *MALAT1* expression levels (mean ± standard deviation) following exposure to the Cy3 gapmer (red squares) and unmodified gapmer U (black squares) were normalized to a negative control (untreated cells). A sigmoidal fit (Cy3: red line, U: black line), including a 95% confidence interval (Cy3: red shaded region, U: gray shaded region), was carried out according to SI Section 3.2. The experiment was conducted in biological duplicates and performed on one occasion.

3.3 Fitting of dose-response curves

The normalized relative *MALAT1* expression (Rel. expr.) *vs.* logarithmized concentration (log*C*) data presented in Supplementary Figs. S22 and S23 were fitted using a Levenberg-Marquardt iteration algorithm to equation:

$$\text{Rel. expr.} = A1 + \frac{(A2 - A1)}{(1 + 10^{((pEC50 - logC)\times p)}}$$

where A1 and A2 are the bottom and top asymptote, respectively, p is the Hill slope, and pEC50 is the sigmoidal inflection point. The fitting was performed using instrumental weighting, *i.e.* data points were weighted by the inverse of the standard deviation (error bars) squared. No bounds or constraints were applied to the fitting parameters. The resulting fitting data is shown in Table S4.

Table S4. Fitting parameters for the *MALAT1* knockdown data presented in Supplementary Fig. S22. Data is presented as fitted value ± standard deviation.

Gapmer	pEC50	A1	A2	p	R^{2a}
U	-5.7 ± 0.2	12.7 ± 11.8	89.6 ± 4.5	-0.83 ± 0.33	0.99
U^w	-5.7 ± 0.2	-4.3 ± 11.2	99.2 ± 7.5	-0.77 ± 0.30	1.00
tC-1	-5.5 ± 0.3	25.1 ± 13.7	80.7 ± 4.4	-1.1 ± 0.79	0.98
tC-2	-5.4 ± 0.3	15.6 ± 21.3	91.3 ± 5.7	-1.0 ± 0.77	0.98
tC-2^w	-5.4 ± 0.3	4.9 ± 21.2	101 ± 7.0	-0.88 ± 0.49	0.99
tC-3	-5.4 ± 0.3	1.7 ± 23.2	101 ± 7.4	-0.84 ± 0.47	0.99
tC^O-1	-5.5 ± 0.4	23.8 ± 16.0	80.3 ± 4.5	-0.92 ± 0.60	0.98
tC^O-2	-5.6 ± 0.3	20.8 ± 12.3	77.7± 4.3	-1.2 ± 0.76	0.98
2CNqA-1	-5.6 ± 0.3	39.1 ± 7.2	80.2 ± 4.1	-1.7 ± 1.9	0.96
2CNqA-2	-5.1 ± 0.4	41.3 ± 18.9	82.2 ± 4.1	-1.6 ± 2.1	0.97
pA-1^{TG}	-5.1 ± 1.5	33.7 ± 69	90.2 ± 5.3	-1.0 ± 1.4	0.80^b
pA-1TA	-5.2 ± 0.2	36.7 ± 11.1	86.4 ± 4.3	-2.0 ± 1.5	0.89^b
pA-2	--^{c}	--^{c}	--^{c}	--^{c}	--^{c}

^{a} Goodness of fit parameter COD (confidence of determination). ^{b} Unsatisfactory fit. ^{c} Unable to fit data.

3.4 Flow cytometry

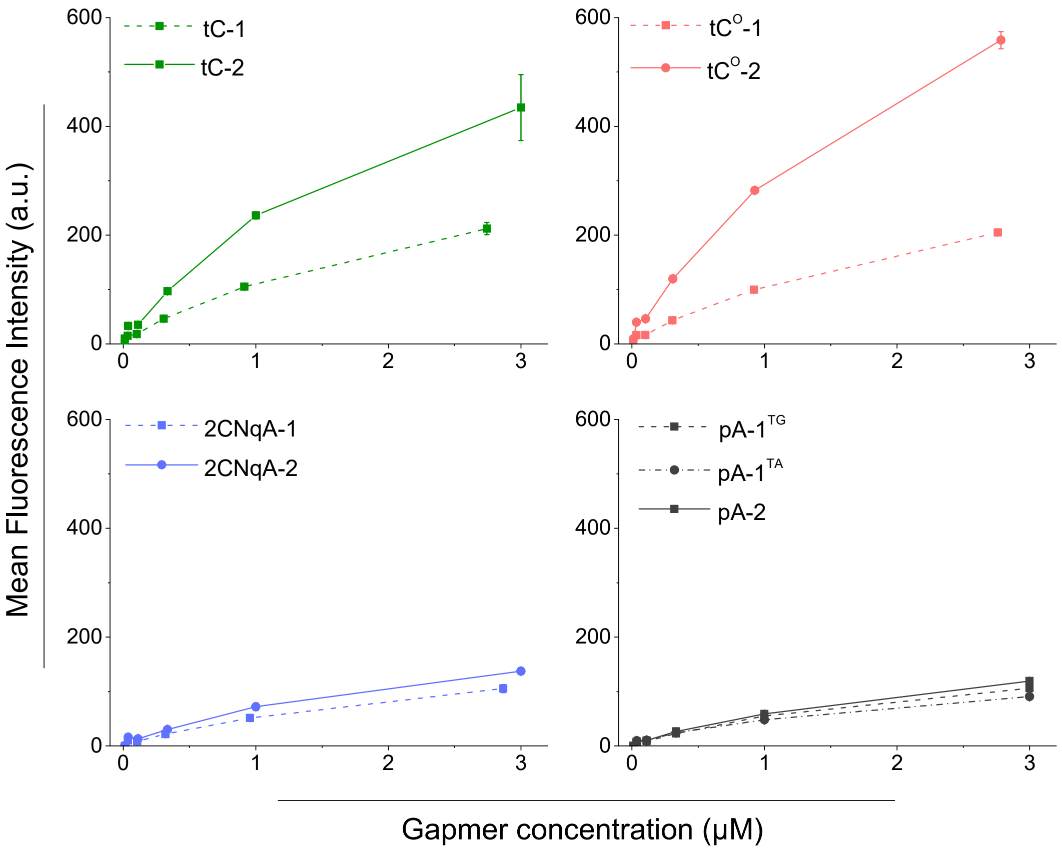

Figure S24. Dose-dependent gapmer uptake in HEK 293T cells after 24 h exposure, evaluated using flow cytometry. This is the non-normalized equivalent to the data shown in Fig. 6A, after subtracting the corresponding MFI from the negative control, MFI(U), which ranged between 95–105 a.u. and 130–142 a.u., respectively, for the two biological replicates.

3.5 Adjustment for filter spectra and emission spectrum in the flow cytometry

Fluorescence intensity is typically directly proportional to excitation light intensity, fluorophore brightness, and concentration. Therefore, given that all wavelengths of the sample emission are uniformly detected, and (like in this work) identical excitation- and detector settings are used for all samples, the relative concentrations of the FBA gapmers can be calculated by simply dividing the detected fluorescence intensity by the gapmer brightness at the excitation wavelength. However, since all emission wavelengths are not detected in our setup and the emission spectra of the FBAs are different, a spectral correction factor (SCF) that represents the fraction of the gapmer emission transmitted through the instrument optical system, must be included in the calculation. In the flow cytometer instrument used in this work, the emitted light from the cells is directed towards the detectors using a series of long pass dichroic mirrors (DM) which, depending on the emission wavelength, either reflect the incident light, or transmit it to a detector. By successively reflecting the light using DMs with different cut-off wavelengths, the emission is sorted into channels based on emission wavelength. For each channel there is an option to further filter the light by inserting a band pass (BP) filter. The relative uptake referred to in this work (Fig. 6) is calculated according to:

$$\text{Relative uptake} = \frac{\text{MFI} - \text{MFI(U)}}{\text{B} \times \text{SCF}}$$

where the MFI and MFI(U) are the uncorrected mean fluorescence intensity (arbitrary unit) for the FBA-labeled gapmers and reference gapmer U, respectively, B is the brightness ($M^{-1}cm^{-1}$) of the single-stranded gapmer at 405 nm, and SCF is a unitless spectral correction factor denoting the fraction of gapmer emission that is transmitted through the optical system to the detector. SCF for each gapmer can be calculated using the emission spectrum of the gapmer and the absorption spectra of all filters in its light path. The emission of the tC gapmers is collected using a different filter combination compared to the other gapmers due to the tC emitting at longer wavelengths. Therefore, the tC emission is reflected on DM(750 nm), DM(690 nm), DM(630 nm),

and DM(595 nm) and transmitted through DM(505 nm) and BP(525/50 nm) before reaching the detector. For all other gapmers, the light is reflected on DM(750 nm), DM(690 nm), DM(630 nm), DM(595 nm), and DM(505 nm) and transmitted through BP(450/50 nm) before hitting the detector. The spectral correction factor for the tC- and non-tC gapmers, respectively, are thus calculated according to:

$$SCF_{tC} = \sum_{\lambda}\left(I(\lambda) \times \prod_{i}\left[1 - \frac{1}{10^{A_i^{DM}(\lambda)}}\right] \times \frac{1}{10^{A_{505}^{DM}(\lambda)}} \times \frac{1}{10^{A_{525}^{BP}(\lambda)})}\right)$$

$$SCF_{non-tC} = \sum_{\lambda}\left(I(\lambda) \times \prod_{k}\left[1 - \frac{1}{10^{A_k^{DM}(\lambda)}}\right] \times \frac{1}{10^{A_{450}^{BP}(\lambda)}}\right)$$

where I(λ) is the emission spectrum of the FBA-labeled gapmer (normalized to *area* = 1), $A^{DM}(\lambda)$ is the absorption spectra of the dichroic mirrors, and $A^{BP}(\lambda)$ is the absorption spectrum of the applied bandpass filter. The *i* indexing corresponds to the tC light path: *i* = 750, 690, 630, and 595 while the *k* indexing corresponds to the non-tC light path: *k* = 750, 690, 630, 595, and 505. In this analysis, it is assumed that the reflectance of the DMs is 1-transmittance, *i.e.* that all photons interacting with the DM are either transmitted or reflected (no losses). This results in the following SCFs: tC-1: 0.288, tC-2: 0.286, tC^{O}-1: 0.321, tC^{O}-2: 0.318, 2CNqA-1: 0.355, 2CNqA-2: 0.347, pA-1^{TG}: 0.286, pA-1TA: 0.281, pA-2: 0.300.

3.6 Cytotoxicity

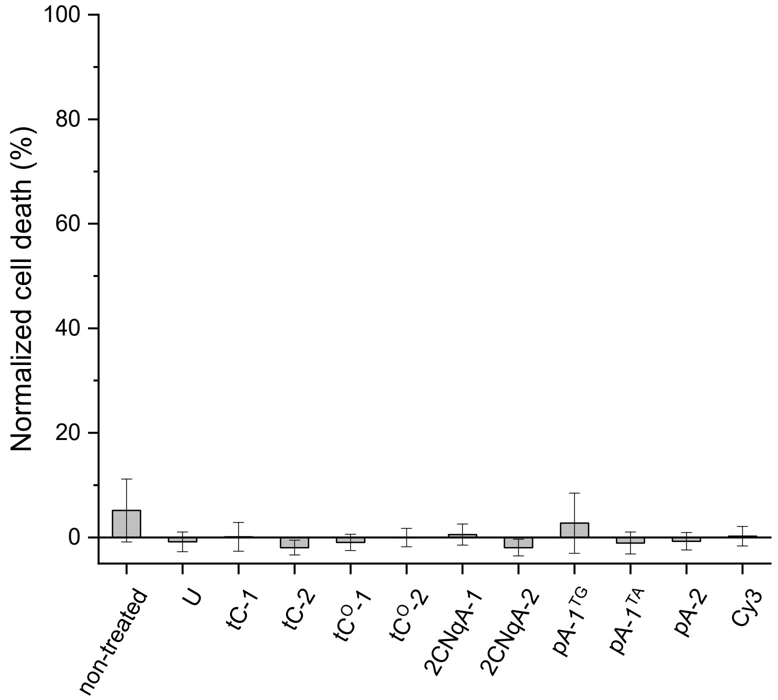

Figure S25. Cytotoxicity of the gapmers was evaluated using the CyQUANT™ LDH cytotoxicity assay in HEK 293T cells after exposure to 3 µM gapmers for 24 h. Normalized fraction of cell death (gray bars, mean ± standard deviation) was calculated by dividing the LDH concentrations in the supernatant by the corresponding values from lysed non-treated cells (100% ± 26% cell death).

3.7 Confocal microscopy

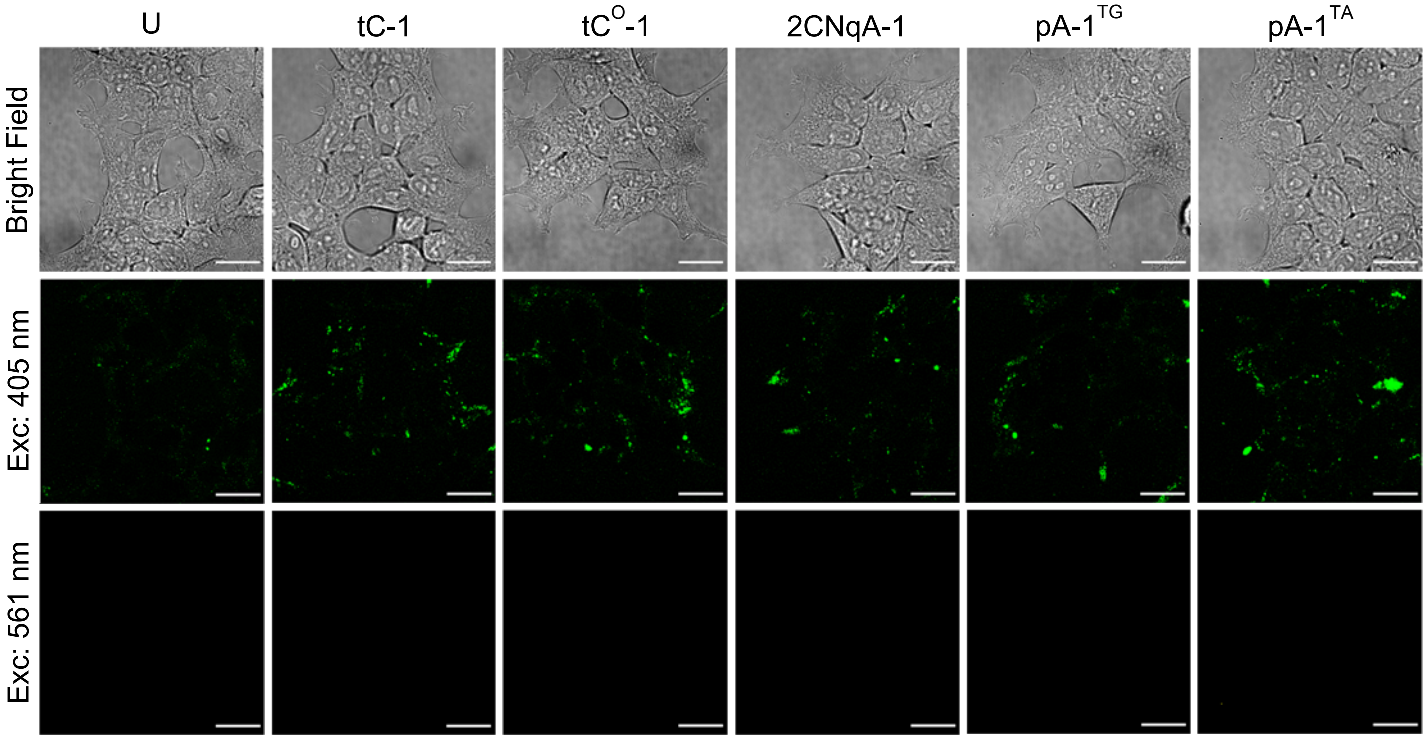

Figure S26. Confocal microscopy images of live HEK 293T cells exposed to unformulated gapmers (3 μM) for 24 h. Samples were excited at 405 nm (emission: 407–700 nm) for FBA detection and at 561 nm (emission: 563–700 nm) for Cy3 detection. Scale bars are 20 μm.

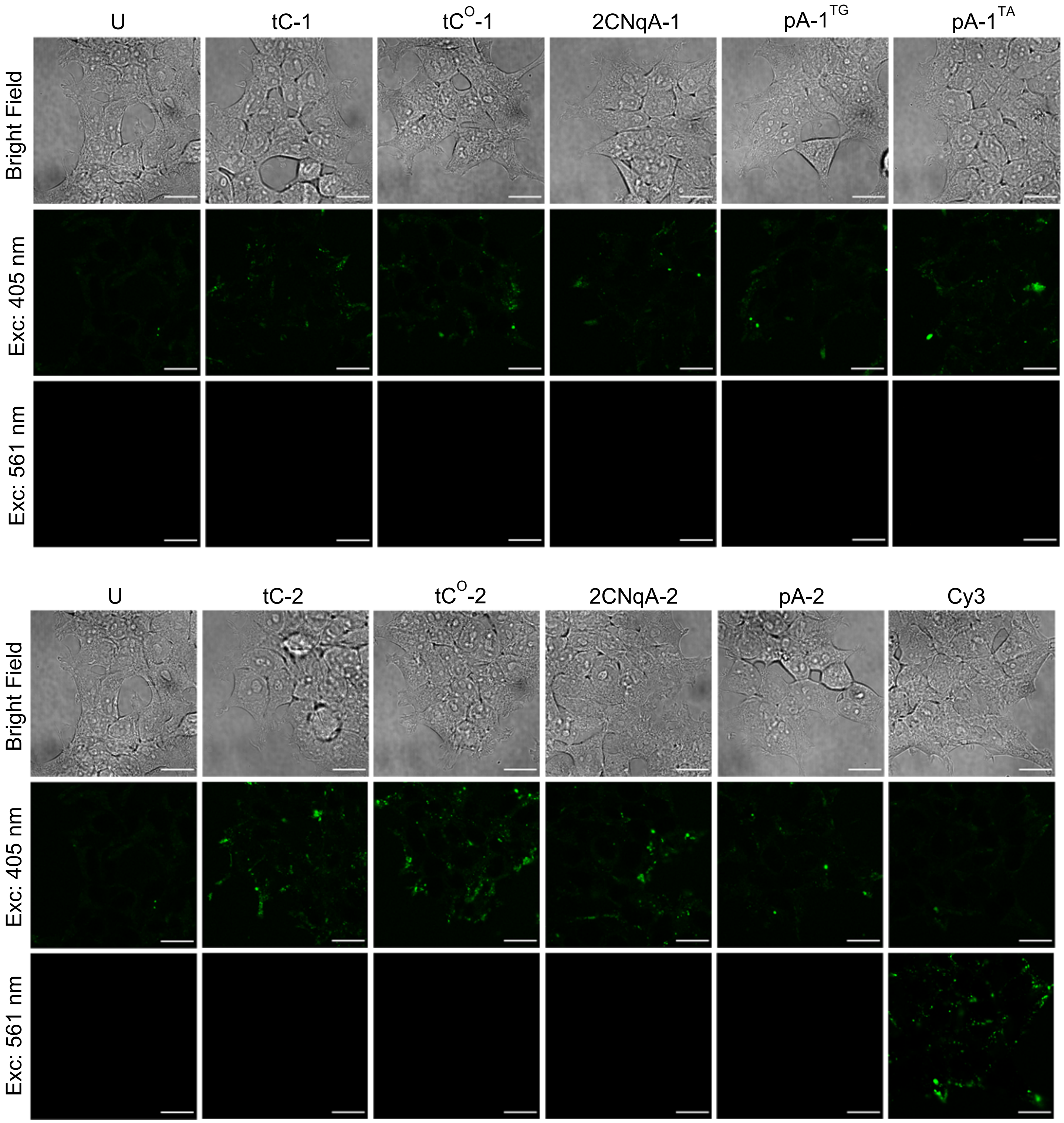

Figure S27. Raw confocal microscopy images of live HEK 293T cells exposed to unformulated gapmers (3 μM) for 24 h. Samples were excited at 405 nm (emission: 407–700 nm) for FBA detection and at 561 nm (emission: 563–700 nm) for Cy3 detection. Scale bars are 20 μm.
